# Lead Compounds from Mangrove-Associated Microorganisms

**DOI:** 10.3390/md16090319

**Published:** 2018-09-07

**Authors:** Elena Ancheeva, Georgios Daletos, Peter Proksch

**Affiliations:** Institute of Pharmaceutical Biology and Biotechnology, Heinrich-Heine-University, Universitaetsstrasse 1, 40225 Düsseldorf, Germany; elena.ancheeva@uni-duesseldorf.de

**Keywords:** mangrove microorganisms, bioactive natural products, endophytes, drug leads

## Abstract

The mangrove ecosystem is considered as an attractive biodiversity hotspot that is intensively studied in the hope of discovering new useful chemical scaffolds, including those with potential medicinal application. In the past two decades, mangrove-derived microorganisms, along with mangrove plants, proved to be rich sources of bioactive secondary metabolites as exemplified by the constant rise in the number of publications, which suggests the great potential of this important ecological niche. The present review summarizes selected examples of bioactive compounds either from mangrove endophytes or from soil-derived mangrove fungi and bacteria, covering the literature from 2014 to March 2018. Accordingly, 163 natural products are described in this review, possessing a wide range of potent bioactivities, such as cytotoxic, antibacterial, antifungal, *α*-glucosidase inhibitory, protein tyrosine phosphatase B inhibitory, and antiviral activities, among others.

## 1. Introduction

Mangrove (mangal) communities represent a coastal habitat located in tropical and subtropical intertidal estuarine zones, occurring in 112 countries, and mostly attributed to latitudes between 30° N and 30° S [1]. Special ecological conditions of mangroves include relatively high tidal range, high average temperature with little seasonal fluctuation, high salinity, strong winds, and muddy anaerobic or sandy soil [1,2,3]. The flora of these communities includes the so-called “exclusive” or true mangroves as well as “nonexclusive” mangrove species that inhabit other terrestrial or aquatic ecosystems (semi-mangrove or mangrove associates). Tomlinson defines true mangroves by several criteria, including exclusive occurrence in mangrove ecosystem, morphological adaptations (e.g., aerial roots and viviparous, water-dispersed propagules), physiological mechanisms for salt exclusion/excretion, as well as taxonomical distinctness from terrestrial species (at least at the generic level), albeit all these characteristics are not necessary to be present among one plant species [1]. One of the main extreme habitat factors that influences mangroves is high salinity, resulting in the following specific leaf traits and osmotic properties of true mangroves: lower specific leaf area, higher succulence, lower K^+^/Na^+^ ratio, higher Na^+^ and Cl^−^ contents, and hence higher osmolality in contrast to semi-mangrove plants [4]. Since mangrove ecosystems can be viewed as an extreme environment, which demands various morphological and physiological adaptations of inhabiting species, the biosynthetic potential of the latter to produce a distinct array of novel chemical entities is apparent. Thus, the mangrove ecosystem is considered as an attractive biodiversity hotspot that is intensively investigated in the hope of discovering new structural scaffolds, including those with medicinal applications. Indeed, studies on secondary metabolites from mangrove plants in the past were found to be quite fruitful, and gave rise to new carbon frameworks accompanied by pronounced biological activities [2]. Notable recent examples of bioactive derivatives from mangroves include the tetranortriterpenoids xylogranatins A–D [5] and krischnadimer A [6], as well as the triterpenoid paracaseolin [7] possessing potent cytotoxic activity toward cancerous cells, the limonoids krishnolides A–D with anti-HIV activity [8], and thaixylomolin B with anti-inflammatory properties [9]. Furthermore, secondary metabolites from typical mangrove plants of the genera *Xylocarpus*, *Avicennia*, *Rhizophora*, and *Bruguiera* significantly enriched our current knowledge of the chemistry of complex tetranorterpenoids (limonoids), di- and triterpenoids, and iridoids, among others [2,10].

**Figure 1:**
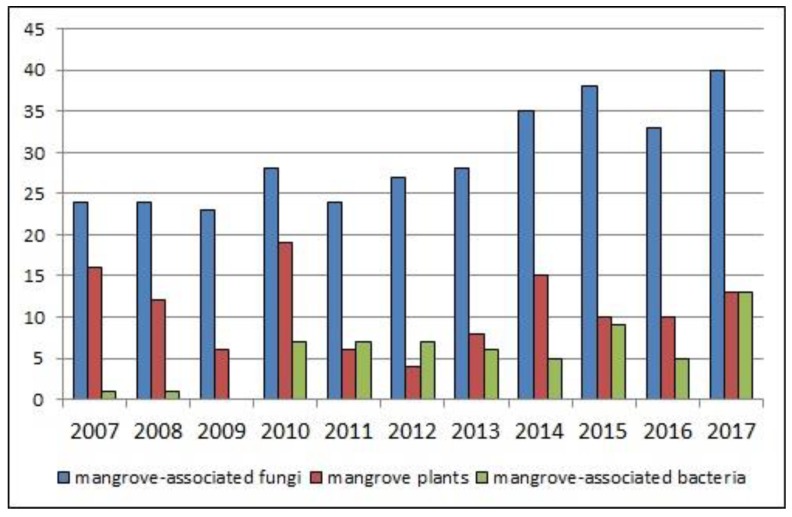
Number of publications describing new and/or bioactive mangrove-associated secondary metabolites covering the period 2007–2017. Source: MarinLit database and series of annual reviews by Blunt et al. in *Natural Product Reports* [13,14]. Articles on mangrove-associated fungi and bacteria include both ecological groups, plant- and soil-derived microorganisms.

Mangrove-associated microorganisms, including fungal and bacterial endophytes, as well as microbes derived from soil samples, have likewise drawn the attention of natural product researchers. Remarkable discoveries of bioactive xyloketals with unprecedented structures from *Xylaria* sp. [11] and salinosporamide A from the mangrove bacterium *Salinospora tropica* [12] in the early 2000s indicated a great potential for drug discovery and inspired further studies on microbes derived from mangroves. Intensive investigation of the latter resulted in more than 350 publications that appeared during the last ten years, focusing on natural product chemistry, bioactivity, biotechnology, and chemical synthesis of their bioactive metabolites, thus demonstrating the potential of mangrove-associated microbes as a prolific source of lead compounds [13]. Particularly, over the last decade, research on these microbes resulted in characterization of almost 1000 new metabolites, among them, ~850 derived from fungi (the majority of them obtained as endophytes), and ~120 from bacteria [14]. Thus, the overall trend in the number of experimental articles dedicated to description of new/bioactive mangrove-derived metabolites over the past ten years, remains promising (Figure 1) [13].

Attempts to estimate fungal and bacterial diversity from soil samples collected in mangroves of Saudi Arabia, China, Brazil, and India showed a high diversity of associated microbes [15,16,17,18,19]. Interestingly, a study directed towards the estimation of bacterial diversity, utilizing 16S rRNA sequencing, demonstrated that the bacterial community of pristine mangrove sediments contains species that are mostly unrelated to known bacteria [19]. Moreover, analysis of culturable fungal endophytes collected in Brazil indicated that among fungal isolates some species could not be classified within any known genera [20]. These investigations, together with promising results from studies on mangrove-associated secondary metabolites, demonstrate this unique ecosystem as a rich reservoir of natural product diversity that could be utilized in the exploration of new drug leads.

Advances in natural products derived from mangrove actinomycetes were discussed by Xu et al. (up to 2013) [21]. Bioactive natural products from mangrove-associated microorganisms, their chemical features and biosynthetic aspects, covering the time span of 2011–2013, were summarized by Jing Xu [22]. In this review, we highlight selected examples (studies in which at least one compound exhibits IC_50_ (/MIC) values less than 10 μM (/10 μg/mL) and/or comparable or higher activity than that of the positive control in the respective bioassay) of recently described microbial bioactive compounds of mangrove origin. The time frame covered in this paper extends from 2014 to March 2018. The articles selected for the review contain natural products with mechanism-of-action studies and/or compounds with pronounced activity. The natural products described herein are grouped according to the microorganism ecological source into endophytes and those derived from soil, the latter group including studies on compounds of fungal or bacterial origin.

## 2. Bioactive Compounds from Mangrove-Associated Microorganisms

### 2.1. Bioactive Compounds from Endophytic Fungi

#### 2.1.1. Cytotoxic Compounds

**Figure 2:**
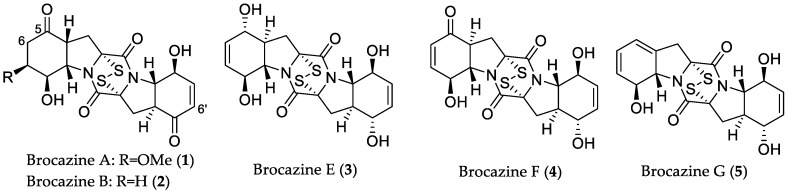
Chemical structures of **1**–**5**.

Meng et al. successfully isolated twenty-one new compounds from a single fungal strain *Penicillium brocae* MA-231, obtained from fresh tissue of the marine mangrove plant *Avicennia marina* (Hainan Island, China) using different types of media for cultivation of this fungus [23,24,25,26]. The first fermentation on potato-dextrose broth (PDB) medium afforded six new disulfide-bridged diketopiperazines brocazines A–F [23]. Among them, brocazines A (**1**), B (**2**), E (**3**), and F (**4**) (Figure 2) displayed cytotoxicity against a panel of human tumor cell lines, including Du145 (human prostate cancer), HeLa (cervical cancer), HepG2 (liver cancer), MCF-7 (breast adenocarcinoma), NCI-H460 (non-small-cell lung cancer), SGC-7901 (gastric cancer), SW1990 (pancreatic adenocarcinoma), SW480 (colon cancer), and U251 (glioblastoma) with IC_50_ values in the range from 0.89 to 12.4 μM, whereas brocazines C and D did not show activity, indicating that the presence of two double bonds at positions C-6 and C-6′ or of one double bond at C-6/6′ in conjugation with a keto group (at C-5/5′), plays an important role in the cytotoxicity of these metabolites. In a further study, chemical investigation of the extract from the fungus grown in Czapek medium showed induction of new natural products, among them, the bisthiodiketopiperazine derivative brocazine G (**5**) (Figure 2) that was active against A2780 (human ovarian cancer) cells that are either sensitive (sens) or resistant (CisR) to the cytostatic drug cisplatin [24]. Interestingly, compound **5** displayed potent activity to both cell lines with IC_50_ values of 664 and 661 nM, respectively. In addition, **5** showed strong activity against *Staphylococcus aureus* with an MIC value of 0.62 μM (0.25 μg/mL), stronger than that of the positive control chloromycetin (MIC = 1.55 μM/0.5 μg/mL). Moreover, other studies on this fungus yielded a series of antibacterial compounds (**20**–**26**) that are described in Section 2.1.2.

**Figure 3:**
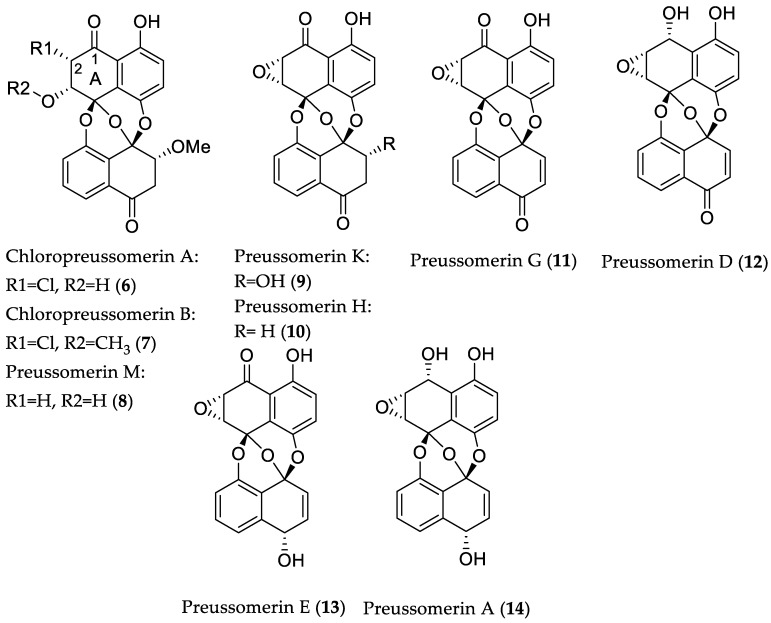
Chemical structures of **6**–**14**.

The endophytic fungus *Lasiodiplodia theobromae* ZJ-HQ1, isolated from healthy leaves of the marine mangrove *Acanthus ilicifolius* (Guangdong Province, China), afforded two new chlorinated preussomerins (**6** and **7**) along with nine known analogs [27]. Interestingly, **6**, **7**, and the known compounds **8**–**14** (Figure 3), exhibited cytotoxicity against a panel of human cancer cell lines, including A549 (lung adenocarcinoma), HepG2, MCF-7, HeLa, and HEK 293T (embryonic kidney) cells, with IC_50_ values ranging from 2.5 to 83 μM. Structure–activity relationship analyses revealed that a ketone group at C-1 and/or a chlorine group at C-2 in ring A are favorable substituents for cytotoxicity of preussomerins. Furthermore, compounds **6**, **7**, **10**, **11**, **13**, and **14** showed activity toward *S. aureus* with MIC values between 4.4 and 35.9 μM (1.6 and 13 μg/mL). However, the respective compounds were found to be inactive against a panel of Gram-negative bacteria, including *Escherichia coli*, *Pseudomonas aeruginosa*, and *Salmonella enteritidis*, thus suggesting their selective inhibitory activity against Gram-positive bacteria, which is probably connected with the exerted cytotoxicity of these metabolites (Figure 3) [27].

**Figure 4:**
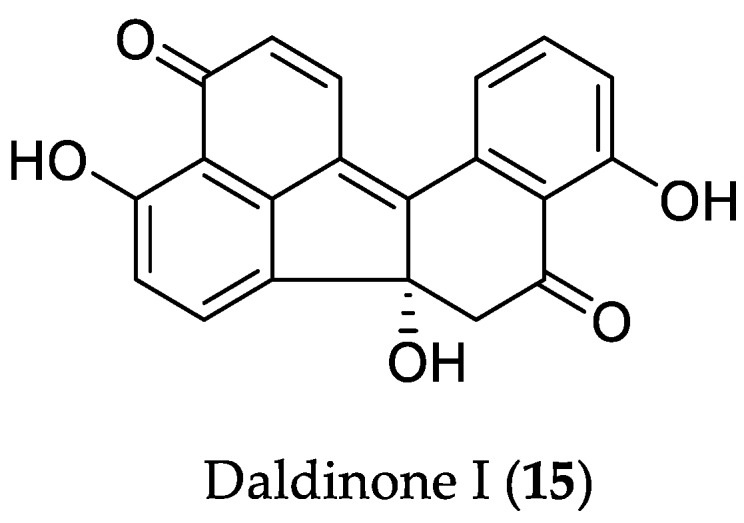
Chemical structure of **15**.

The endophyte *Annulohypoxylon* sp. CA-2013, isolated from the mangrove plant *Rhizophora racemosa*, collected in Cameroon*,* led to the characterization of new benzo[*j*]fluoranthene-based congeners, daldinones H–J [28]. All compounds were tested for their cytotoxicity, and daldinone I (**15**) was shown to be the most active derivative (Figure 4). Interestingly, **15** was shown to be an artefact formed from daldinone H through intramolecular dehydration. Compound **15** exhibited strong to moderate cytotoxicity against adult lymphoblastic leukemia T cells (Jurkat J16) and Burkitt’s lymphoma B lymphocytes (Ramos), with IC_50_ values of 14.1 and 6.6 μM, respectively. Mechanism-of-action studies showed that **15** activates caspases, and subsequently induces apoptosis in Ramos cells with a rapid kinetic profile, comparable to that of the potent known apoptosis inducer staurosporine. Treatment of caspase-9-deficient and caspase-9-reconstituted Jurkat cells with **15** revealed that its pro-apoptotic effect is connected with caspase-9-dependent intrinsic (mitochondrial) apoptosis. Moreover, treatment of murine embryonic fibroblasts (MEF) cells possessing high expression level of mCitrine-hLC3B with **15**, and subsequent analysis of the level of LC3 protein (component of the membrane structure of autophagosomes) in cells, indicated that daldinone I (**15**), alone or together with the pan-caspase inhibitor QVD, shows inhibition of autophagy in a caspase-independent manner (Figure 4).

**Figure 5:**
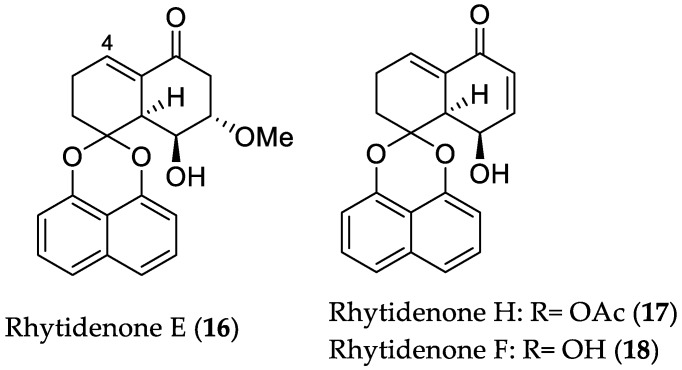
Chemical structures of **16**–**18**.

Investigation of the fungal strain *Rhytidhysteron rufulum* AS21B, isolated from *Azima sarmentosa*, which was collected from a mangrove area in Samutsakhon Province, Thailand, afforded a series of spirobisnaphthalene analogs, among them, compounds **16**–**18** and **40**–**41**, exhibiting cytotoxic and nitric oxide (NO) production inhibitory activities, respectively (see Section 2.1.3). Cultivation of the fungus under slightly acidic conditions (pH = 5) led to a distinct change in its metabolic profile, affording two new spirobisnaphthalenes, rhytidenones G and H (**17**), along with eleven known compounds, among them, rhytidenones E and F (**16** and **18**, respectively) [29]. The production of the latter compound (**18**) was increased 8-fold compared to the culture under normal conditions (pH = 7), allowing its detection and isolation from the culture extract. Compounds **16** and **18** displayed potent activity against Ramos and drug resistant NSCLC H1975 (non-small cell lung cancer) cells, with IC_50_ values in the range between 0.018 and 1.17 μM. Moreover, compound **17** showed selective activity toward the Ramos lymphoma cell line with an IC_50_ value of 0.461 μM. These findings suggest that the C-4 *α*,*β*-unsaturated ketone moiety is essential for the potent cytotoxicity of these metabolites (Figure 5) [29].

**Figure 6:**
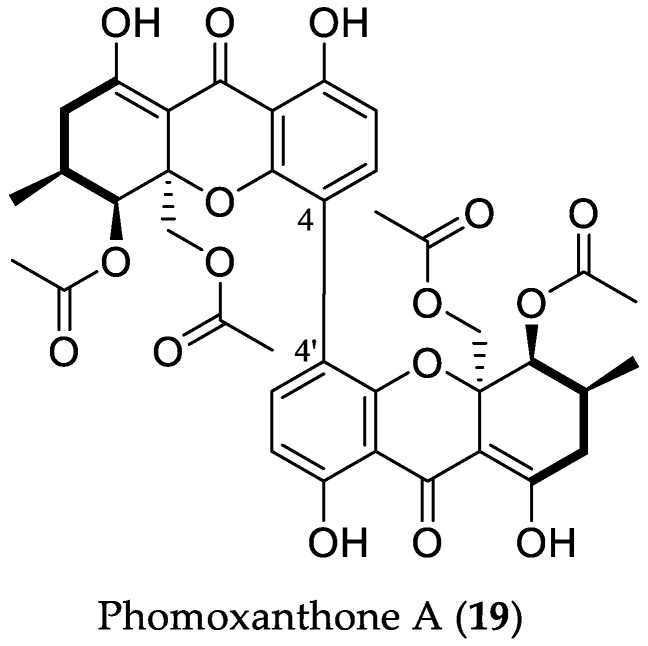
Chemical structure of **19**.

Phomoxanthone A (**19**) is a tetrahydroxanthone dimeric natural product that has attracted significant attention due to its cytotoxic, antibacterial, and antifungal properties. In our research group, this intriguing metabolite was isolated from the endophyte *Phomopsis longicolla* that was derived from the mangrove plant *Sonneratia caesolaris*, collected on Hainan Island, South China (Figure 6) [30,31]. Initial cytotoxicity screening of **19** showed pronounced growth inhibition of cisplatin-sensitive and -resistant cell lines (IC_50_ in the range from 0.7 to 5.2 μM) in our bioassays, confirming the literature data [30]. Preliminary mechanistic investigations revealed caspase activation and proapoptotic activities of **19**. Moreover, potent activation of murine T cells, NK cells, and macrophages after treatment with **19** were observed assuming cell immune stimulation to be part of the biological profile of phomoxanthone A (**19**). Semisynthetic studies revealed that a 4–4′ linkage between the tetrahydroxanthone monomers is favorable for mediating cytotoxicity [30]. The pronounced cytotoxicity of phomoxanthone A (**19**) towards cancer cells encouraged further investigation of its mode of action, through which the apoptotic events in the cells are released. At first, the effect of **19** on cellular Ca^2+^ levels was examined [32]. As a result, the cytosolic Ca^2+^ concentration in Ramos cells treated with **19** was strongly increased, showing a similar pattern to that of the tyrosine phosphatase inhibitor pervanadate; however, **19** did not show any activity when tested against a broad panel of protein kinases. Furthermore, **19** was able to increase the cytosolic Ca^2+^ level in the absence of extracellular Ca^2+^, thus suggesting that the Ca^2+^ increase is caused by direct effects of the compound on the endoplasmic reticulum (ER) or on mitochondria. Co-treatment of the cells with thapsigargin, an inhibitor of the ER CaATPase, and **19** resulted in a higher increase of the Ca^2+^ concentration than that observed for cells treated solely with **19**, thus indicating that the Ca^2+^ origin caused by **19** should be connected with other cell sources of these ions. Indeed, estimation of the effect of **19** on Ca^2+^ using HeLa cells expressing CEPIA (calcium-measuring organelle-entrapped protein indicators) Ca^2+^ probes targeted to the ER or mitochondria confirmed that **19** causes strong and rapid depletion of Ca^2+^ stored in mitochondria. Further comparison of the mitochondrial response after treatment with **19** with effects of the known mitochondrial permeability transition pore (mPTP) inducer ionomycin, and the mPTP inhibitor, cyclosporine A, allowed the conclusion that Ca^2+^ release caused by **19** is largely independent from the mPTP mechanism. Therefore, the mitochondrial Ca^2+^ release is likely to be connected with changes in other mitochondrial ion gradients. Further analysis of the effect of **19** revealed immediate depolarization of the membrane potential ΔΨ_m_, similar to carbonyl cyanide *m*-chlorophenyl hydrazone (CCCP), an inhibitor of oxidative phosphorylation. However, **19** produced only a slight decrease in cellular O_2_ consumption, in contrast to the rapid kinetics by CCCP, caused through the uncoupling of the proton gradient. Moreover, measurement of O_2_ consumption after respiration enhancement by CCCP showed a strong decrease in O_2_ utilization, pointing to the inhibition of cellular respiration and of electron transport chain (ETC) by **19**. Detailed analysis of the effects of **19** on these mitochondrial processes based on comparison with known ETC inhibitors that target different complexes of the ETC and ATP synthase, such as rotenone, thenoyltrifluoroacetone, and antimycin A, suggested that **19** disturbs complex I and II of ETC or interferes with the shuttling of electrons between complex I/II and III. Moreover, **19**, like CCCP, caused stress-induced OPA1 (enzyme essential for regulation of the equilibrium between mitochondrial fusion and mitochondrial fission) cleavage that was dependent on metalloendopeptidase OMA1, and led to cristae disruption, although it was proven that the disruption occurs independently of OMA1. Since mitochondrial fission is also regulated by the dynamin-related protein DRP1, which mediates outer mitochondrial membrane fission, the DRP1-deficient MEF cells were analyzed in the presence of **19**, and the fragmentation was likewise observed. Experiments with dual staining of the mitochondrial matrix and the outer mitochondrial membrane, affected by **19** and CCCP (a positive control for fragmentation), showed that the outer membrane appeared to remain connected. This effect could also be observed in cells deficient for both DRP1 and OPA1, indicating induction of mitochondrial fission by **19** independently of canonical regulators. Finally, a close examination of the mitochondrial ultrastructure by transmission electron microscopy (TEM) revealed that **19** causes OMA1-independent disruption of mitochondrial matrix morphology, complete loss of cristae, and condensation of inner membrane structures at the outer membrane. Taken together, it was shown that the inner, but not the outer, mitochondrial membrane structure rapidly collapses into fragments, leading to cristae disruption, and eventually apoptosis upon treatment with **19**, thus implying that phomoxanthone A (**19**) is a mitochondrial toxin with a novel mode of action, which is distinct from other known ETC inhibitors, OXPHOS uncouplers, and ionophores. Further studies on the identification of the molecular target of **19**, through which mitochondrial Ca^2+^ release and inner mitochondrial membrane fission is induced, will undoubtedly uncover the mechanism of action of this interesting mycotoxin [32].

#### 2.1.2. Antimicrobial Compounds

**Figure 7:**
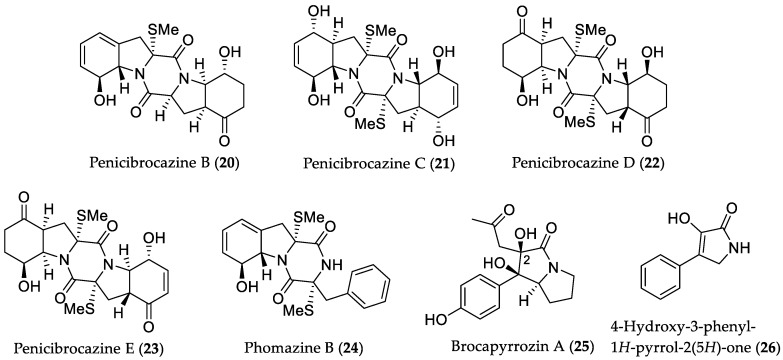
Chemical structures of **20**–**26**.

Subsequent work on the fungal extract of *P. brocae* MA-231 (see Section 2.1.1), cultured on PDB medium, allowed for the characterization of five new sulfide diketopiperazine derivatives, namely, penicibrocazines A–E, and one known analog, phomazine B (**24**) [25]. All compounds were tested for their antimicrobial properties against several human- or plant-pathogenic, as well as marine microorganisms. Penicibrocazines B–D (**20**–**22**) and **24** (Figure 7) showed activity against *S. aureus* with MIC values of 82, 0.55, 17.6, and 0.55 μM (32.0, 0.25, 8.0 and 0.25 μg/mL), respectively, compared to the positive control chloromycetin (MIC = 12.4 μM/4.0 μg/mL). Compound **21** showed strong inhibiting activity against *Micrococcus luteus* with an MIC value of 0.55 μM (0.25 μg/mL), which is higher than that of chloromycetin (MIC = 6.2 μM/2.0 μg/mL). Moreover, derivatives **20**, **22**, penicibrocazine E (**23**) and **24** were active against the plant pathogen *Gaeumannomyces graminis* with MICs 0.64, 17.6, 0.55, and 159 μM (0.25, 8.0, 0.25, and 64.0 μg/mL), respectively, whereas the positive control amphotericin B displayed an MIC value of 17.3 μM (16.0 μg/mL). Evaluation of penicibrocazines A–E against eight tumor cell lines showed no significant cytotoxicity (IC_50_ > 10 μM). Further cultivation of *P. brocae* in liquid Czapek medium yielded four new thiodiketopiperazine alkaloids, penicibrocazines F–I, as well as two new *N*-containing *p*-hydroxyphenopyrrozin derivatives brocapyrrozins A (**25**) and B [26]. Subjected to the same bioactivity assays as in the previous report [25], **25** and the known compound 4-hydroxy-3-phenyl-1*H*-pyrrol-2(5*H*)-one (**26**) (Figure 7) showed strong inhibitory activity against the bacterium *S. aureus* and the fungus *Fusarium oxysporum* with MIC values ranging from 0.41 to 2.85 μM (0.125 to 0.5 μg/mL). These values were equipotent or stronger than those of the positive antibacterial and antifungal controls, chloromycetin (MIC = 1.55 μM/0.5 μg/mL) and zeocin (MIC = 0.35 μM/0.5 μg/mL), respectively. The aforementioned results indicated that the presence of an acetonyl group at C-2 is favorable for the antibiotic activity of brocapyrrozins.

**Figure 8:**
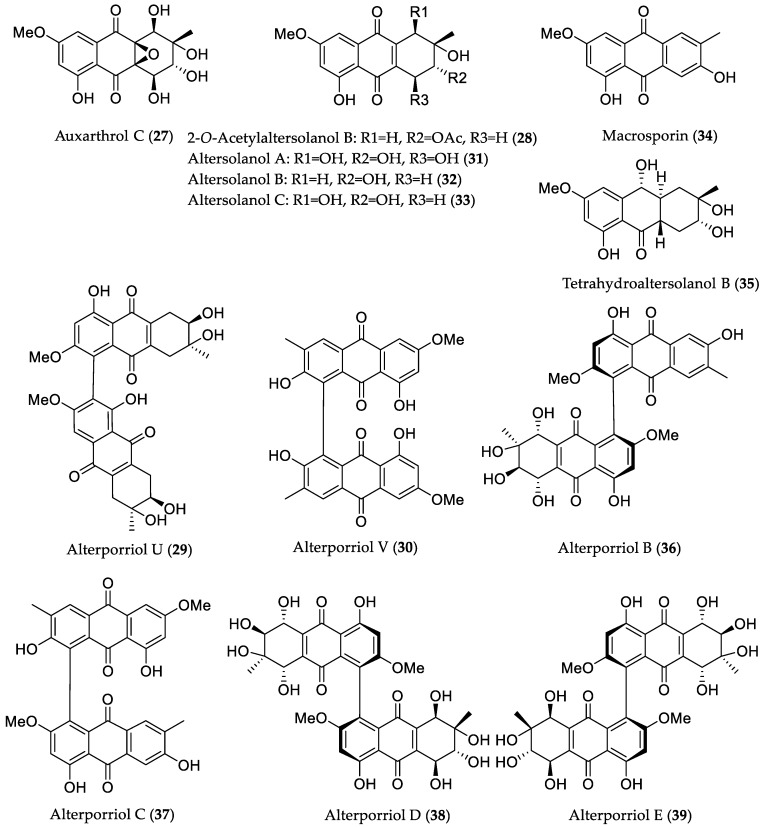
Chemical structures of **27**–**39**.

A fungal strain *Stemphylium* sp. 33231 isolated from the mangrove *Bruguiera sexangula* var. *rhynchopetala* collected from the South China Sea yielded eight new and seventeen known metabolites [33]. Among them, two new anthraquinone derivatives (**27** and **28**), two alterporriol-type anthranoid dimers (**29** and **30**)**,** as well as the known analogs, altersolanols A–C (**31**–**33**), macrosporin (**34**), tetrahydroaltersolanol B (**35**), and alterporriols B (**36**), C (**37**), D (**38**), and E (**39**), were found to possess moderate or weak antibacterial properties when tested against a panel of terrestrial and pathogenic bacteria, such as *Micrococcus tetragenus*, *E. coli*, *Staphylococcus albus*, *Bacillus cereus*, *S. aureus*, *Kocuria rhizophila*, and *Bacillus subtilis* (Figure 8). Metabolites **28**, **31**, **32**, **34**, and **38** were active against at least three bacterial strains, with minimum inhibitory concentration (MIC) values in the range between 2.07 and 10 μM. Compounds **27**, **33**, and **37** exhibited selective activity against *E. coli* (MIC = 9.8 μM), *B. subtilis* (MIC = 8.8 μM), and *S. albus* (MIC = 8.9 μM), respectively, while compounds **29**, **30**, and **36** showed selectivity against *B. cereus* strain with MIC values of 8.3, 8.1, and 7.9 μM, correspondingly. In addition, compound **39** showed antibacterial activity against two tested strains, *B. cereus* (MIC = 2.5 μM) and *E. coli* (MIC = 5.0 μM). All aforementioned metabolites, with the exception of **33** and **35**, were investigated for cytotoxicity against mouse melanoma (B16F10) and A549 cell lines, however, showed no activity (IC_50_ > 10 μM). Moreover, these compounds were found to be inactive when tested for brine shrimp lethality using *Artemia salina*, thus suggesting that their antibacterial activity is not due to general cytotoxicity [33].

#### 2.1.3. Compounds with Inhibitory Activity towards NO Production

**Figure 9:**
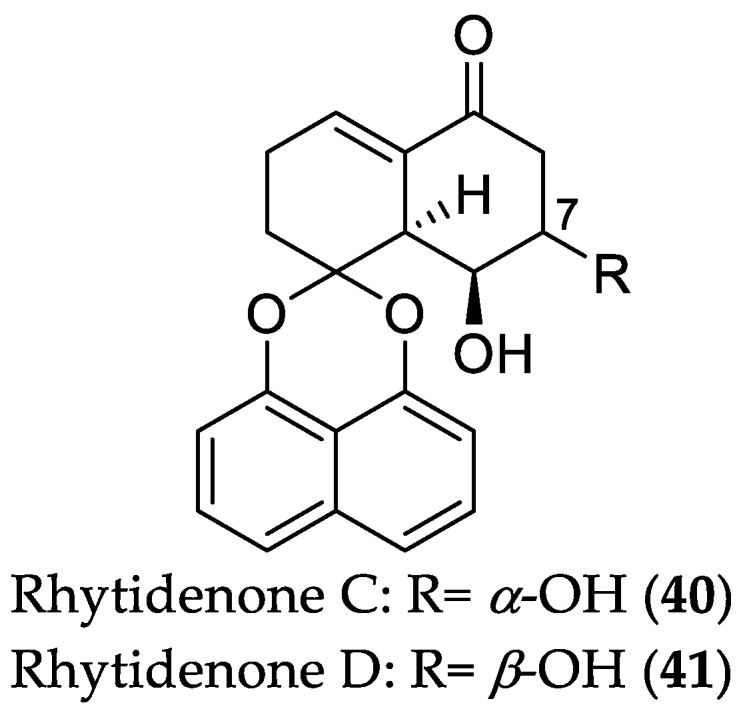
Chemical structures of **40** and **41**.

The endophyte *R. rufulum* AS21B (see Section 2.1.1) afforded six new spirobisnaphthalene derivatives, namely, rhytidenones A–F [34]. Among the isolated compounds, rhytidenone C (**40**) showed the most potent inhibitory effect on NO production in lipopolysaccharide (LPS)-stimulated J774.A1 macrophages with an IC_50_ value of 0.31 μM. On the other hand, the anti-inflammatory activity of rhytidenone D (**41**) was 10-fold lower (IC_50_ = 3.60 μM), indicating that the *α*-orientation of the hydroxy group at position 7 is favorable for the anti-inflammatory activity of these metabolites. Remarkably, **40** and **41** exhibited no cytotoxicity in the respective cells, thus indicating that they are potential leads for the development of anti-inflammatory agents (Figure 9).

**Figure 10:**
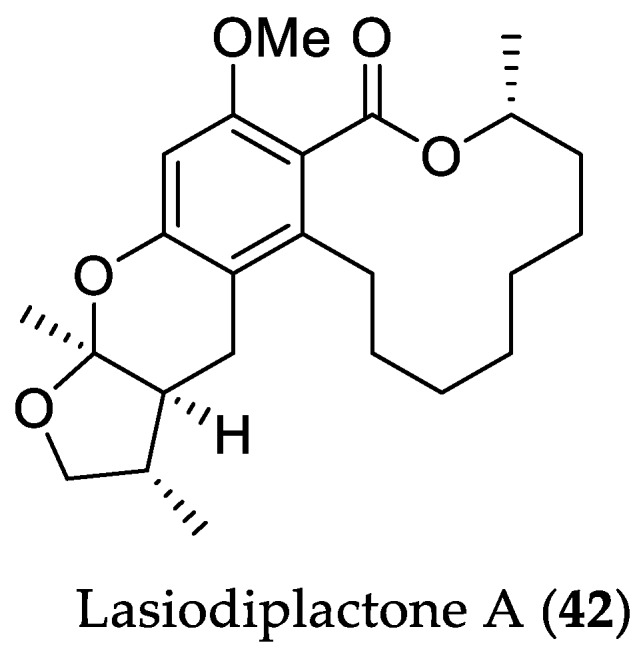
Chemical structure of **42**.

Cultivation of the fungus *L. theobromae* ZJ-HQ1 (see Section 2.1.1) on solid rice media with different salinity (0.3%) led to the isolation of the novel lactone lasiodiplactone A (**42**) with an unusual tetracyclic system, containing a 12-membered *β*-resorcylic acid lactone, a pyran, and a furan ring (Figure 10) [35]. Compound **42** showed potent inhibitory activity toward NO production in LPS-activated RAW264.7 cells with an IC_50_ value of 23.5 μM, comparable to that of the positive control indomethacin (IC_50_ = 26.3 μM). Interestingly, compound **42** did not show any cytotoxicity against LPS-activated RAW264.7 cells up to 100 μM, thus indicating that the NO inhibitory effect of **42** is not due to cytotoxicity. Moreover, compound **42** displayed strong *α*-glucosidase inhibitory activity with an IC_50_ value of 29.4 μM, which was stronger than the clinically used anti-diabetic drug acarbose (IC_50_ = 367 μM).

#### 2.1.4. Compounds with *α*-Glucosidase Inhibitory Activity

**Figure 11:**
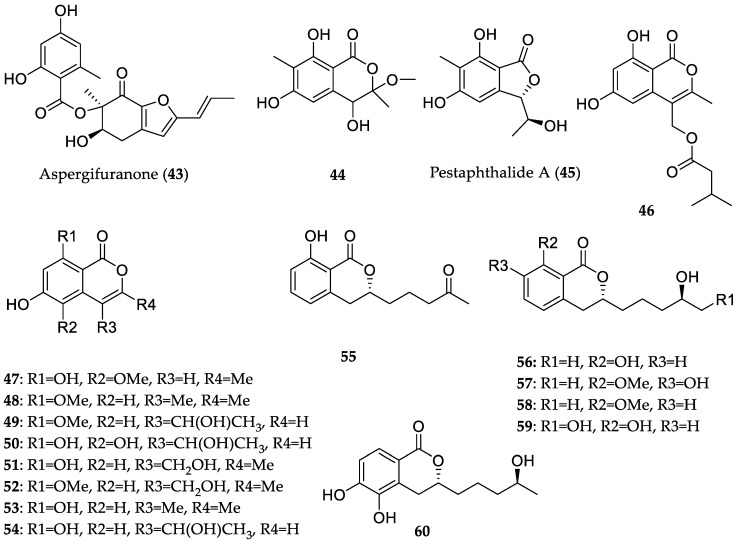
Chemical structures of **43**–**60**.

The endophytic fungus *Aspergillus* sp. 16-5B, obtained from leaves of *Sonneratia apetala* (Hainan Island, China), was investigated, and this resulted in the characterization of polyketides with in vitro *α*-glucosidase inhibitory activity (**43**–**45**) (Figure 11) [36]. Interestingly, the new metabolite aspergifuranone (**43**) showed significant inhibition of enzyme activity, with an IC_50_ value of 9 μM, which is approximately 60-fold more potent than the clinically used *α*-glucosidase inhibitor acarbose (IC_50_ = 554 μM). Moreover, a new isocoumarin derivative (**44**), which was isolated as a racemate, along with the known metabolite pestaphthalide A (**45**), displayed considerable inhibitory activities against *α*-glucosidase with IC_50_ values of 90 and 70 μM, respectively. Subsequent kinetic analysis of the most active compound (**43**) revealed that it exhibits noncompetitive inhibition characteristics. A later study from the same research group led to the isolation of fifteen isocoumarins (**46**–**60**), including six new natural products, which were derived from *Talaromyces amestolkiae* YX1, an endophyte of *Kandelia obovata* (Guangdong Province, China) (Figure 11) [37]. Remarkably, all these derivatives exhibited *α*-glucosidase inhibiting activity, with IC_50_ values ranging from 17.2 to 585.7 μM, which were more potent than acarbose (IC_50_ = 958.3 μM).

#### 2.1.5. Compounds with *Mycobacterium tuberculosis* Protein Tyrosine Phosphatase B (MptpB) Inhibitory Activity

**Figure 12:**
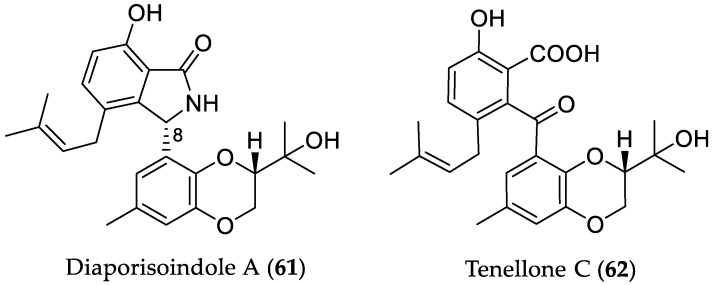
Chemical structures of **61** and **62**.

The endophytic fungus *Diaporthe* sp. SYSU-HQ3, isolated from the mangrove plant *Excoecaria agallocha*, afforded three isoprenylisoindole alkaloids with a rare 1,4-benzodioxan moiety, namely diaporisoindoles A–C, in addition to the known derivative tenellone C (**62**) [38]. Diaporisoindole A (**61**) and **62** (Figure 12) showed strong inhibitory activity toward MptpB, with IC_50_ values of 4.2 and 5.2 μM, respectively (positive control oleanolic acid; IC_50_ 22.1 μM). Interestingly, the co-isolated diastereomer diaporisoindole B, possessing an 8*R* configuration, displayed no activity, indicating that the *S* configuration at C-8 is essential for the MptpB inhibitory effect of **61**. In order to get further insights into the mode of action of these compounds, enzyme kinetic analysis was performed. The results showed that **61** acted as an uncompetitive inhibitor, whereas **62** as a competitive inhibitor. Moreover, compounds **61** and **62** showed no inhibitory activity against protein tyrosine phosphatase 1B (PTP1B) at a concentration of 200 μM, suggesting that they are selective MptpB inhibitors, and thus potential leads for anti-TB investigation.

**Figure 13:**
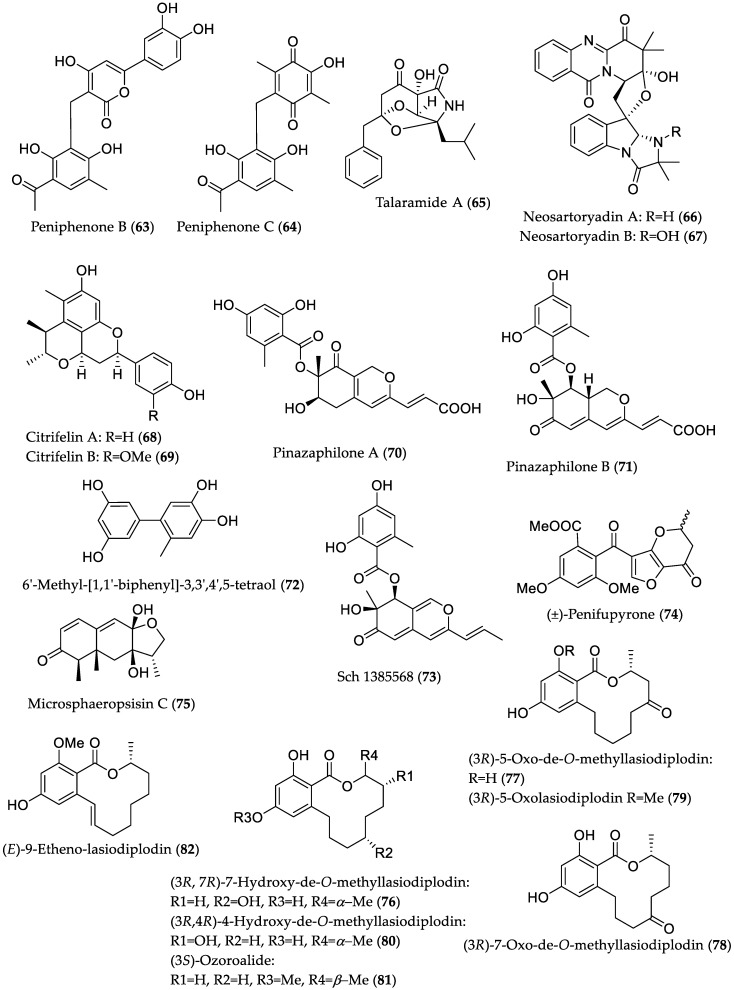
Chemical structures of compounds with MptpB- (**63** and **64**), mycobacterial PknG (**65**), anti-infective (**66**–**69**) and *α*-glucosidase (**70**–**82**) inhibitory activities derived from mangrove endophytic fungi.

**Figure 14:**
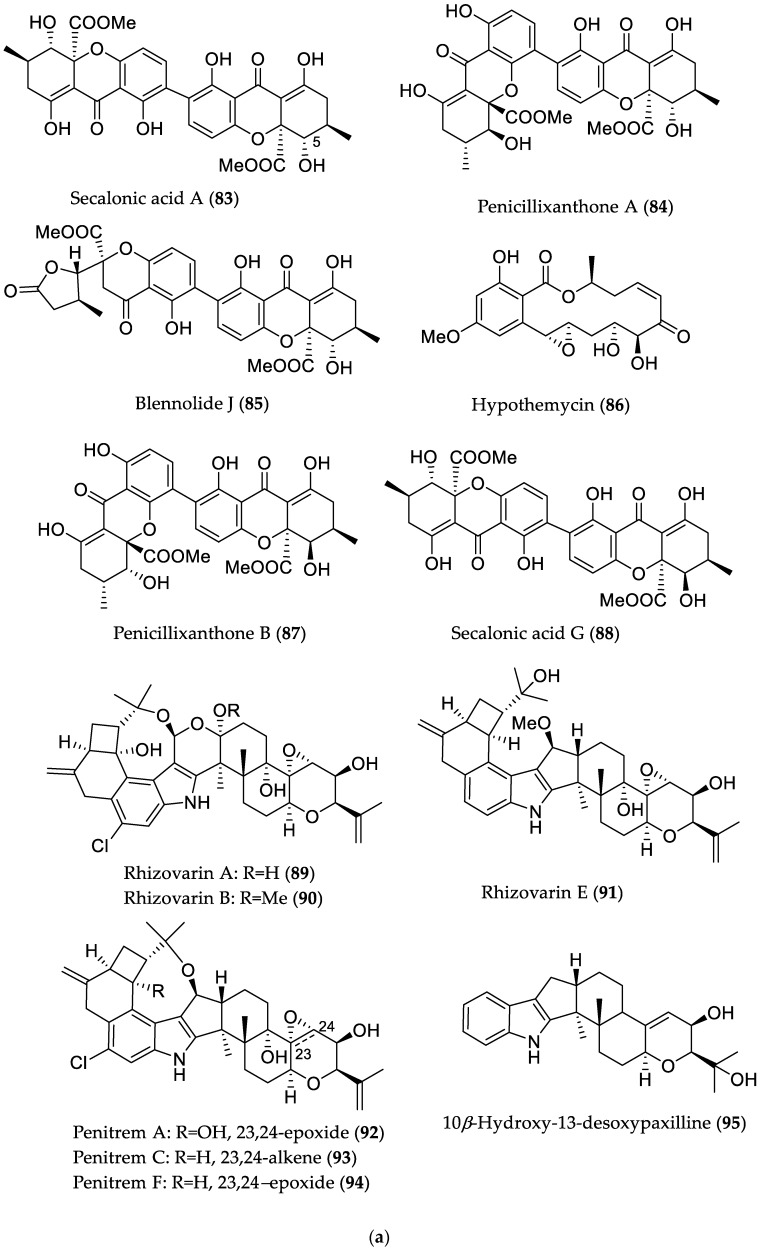

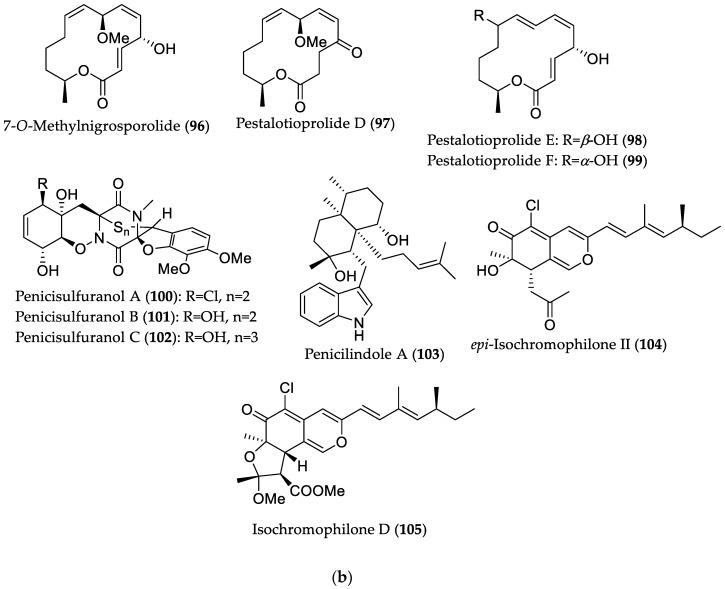
(**a**) Chemical structures of compounds with cytotoxic activity (**83**–**95**) derived from mangrove endophytic fungi. (**b**) Chemical structures of compounds with cytotoxic activity (**96**–**105**) derived from mangrove endophytic fungi.

Further bioactive metabolites derived from mangrove endophytes that could serve as potential lead structures are presented in Table 1, and Figure 13 and Figure 14.

### 2.2. Bioactive Compounds Derived from Fungi Originating from Mangrove (Rhizosphere) Soil/Sediment Samples

#### 2.2.1. Cytotoxic Compounds

**Figure 15:**
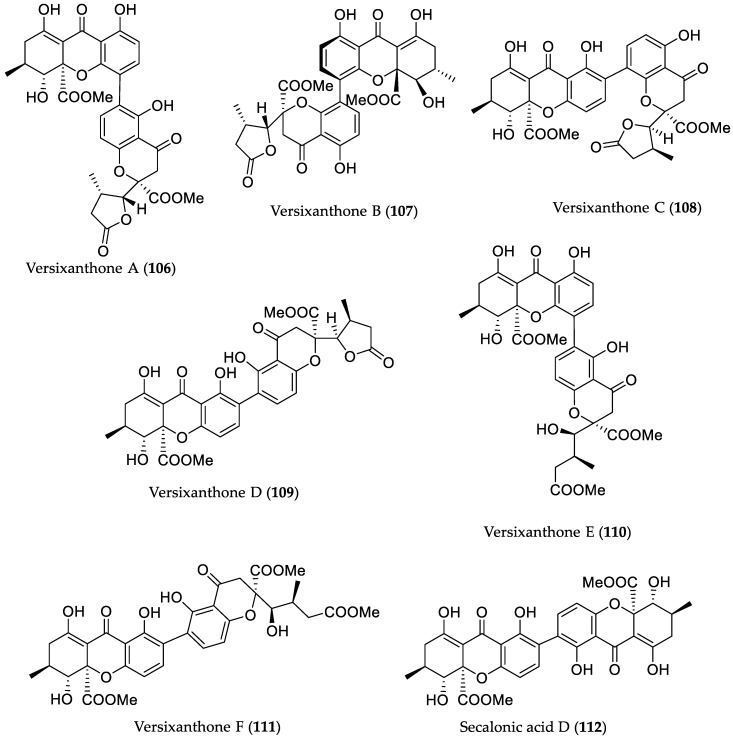
Chemical structures of **106**–**112**.

The fungal strain *Aspergillus versicolor* HDN1009 derived from mangrove soil that was collected in Guangzhou, China yielded six unusual heterogeneous dimers, versixanthones A–F (**106**–**111**) possessing a tetrahydroxanthone unit and a biogenetically-related chromanone monomer coupled via a biaryl linkage, as well as a known compound, secalonic acid D (**112**) [51]. All compounds were tested for cytotoxicity toward HL-60, K562 (myelogenous leukemia), A549, H1975, MGC-803 (human gastric cancer), HO8910 (ovarian cancer), and HCT-116 (colorectal carcinoma) cell lines. Interestingly, compounds **106**–**108** showed activity against at least two cell types, with IC_50_ values between 2.6 and 25.6 μM, and derivatives **109**–**112** exhibited cytotoxicity against at least five cancer lines with IC_50_ values ranging between 0.7 and 21 μM (Figure 15). Remarkably, among all new compounds, only **110** revealed topoisomerase I inhibitory activity, as was previously shown for the known derivative secalonic acid D (**112**) [51].

**Figure 16:**
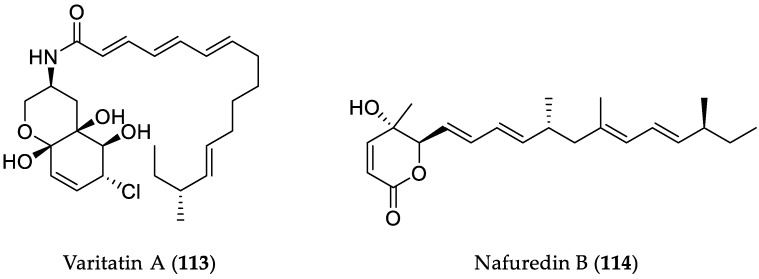
Chemical structures of **113** and **114**.

Addition of the DNA methyltransferase inhibitor 5-azacytidine to the fungus *Penicillium variabile* HXQ-H-1, isolated from the mangrove rhizosphere soil collected on the coast of Fujian Province, China, led to alteration of the fungal metabolome, yielding a highly modified fatty acid amide, varitatin A (**113**) (Figure 16) [52]. Interestingly, **113** exhibited activity against the HCT-116 cell line with an IC_50_ value of 2.8 μM. Moreover, it inhibited 50% and 40% of the protein tyrosine kinases PDGFR-*β* and ErbB4, respectively, at a concentration of 1 μM, suggesting that the cytotoxicity of **113** is probably exerted due to its protein kinase inhibitory activity. Subsequent mixed fermentation of this mangrove fungal strain with the deep-sea-derived fungus *Talaromyces aculeatus* (collected at a depth of 3386 m, Indian Ocean) afforded four novel polyketides, penitalarins A–C, containing an unusual 3,6-dioxabicyclo[3.1.0]hexane, in addition to nafuredins A and B (**114**) (Figure 16), which were not detected in the axenic fungal cultures under the same conditions [53]. Compound **114** showed cytotoxicity against a panel of human cancer cell lines (HeLa, K562, HCT-116, HL-60, A549, and MCF-7), with IC_50_ values in the range from 1.2 to 9.8 μM, whereas penitalarins A–C and nafuredin A proved to be inactive.

**Figure 17:**
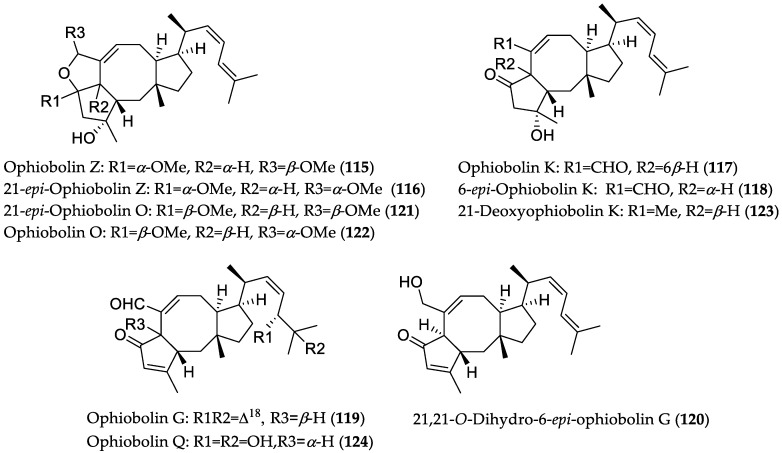
Chemical structures of **115**–**124**.

In 2009, a study on the chemical constituents of the solid-phase culture of the fungus *Aspergillus ustus* 094102 (from *Bruguiera gymnorrhiza* collected in Hainan Province, China) afforded a series of cytotoxic drimane sesqui- and meroterpenoids [54]. To further investigate the biosynthetic capacity of *A. ustus*, this fungus was cultured in both liquid and on solid media to obtain extracts enriched with ophiobolin derivatives. Eventually, the chromatographic work-up of both extracts yielded seven new and eleven known ophiobolin congeners [55]. Interestingly, ophiobolins were only produced during static cultivation, but not in the shaking mode. In addition, the metabolic profile of liquid cultivation under static condition was investigated at different times/days; however, no significant effect on the production of ophiobolins was found. Compounds **115**–**124** (Figure 17) showed cytotoxicity against the human gemcitabine-resistant G3K (pancreatic cancer) cell line, MCF-7, MD-MBA-231 (triple-negative breast cancer) cells, MCF-7/Adr (adriamycin-resistant human breast cancer) cell line, MCF-10A (nontumorigenic breast epithelial cell line), A549, and HL-60 cells, with IC_50_ values in the range from 0.6 to 9.5 μM. Among the tested compounds, 21-*epi*-ophiobolin O (**121**) was found to be the most active analog, with IC_50_ values of 0.6 and 0.8 μM toward the A549 and HL-60 cell lines, respectively, thus suggesting that the 2,5-dimethoxyl-2*H*,3*H*,5*H*-furan moiety is a key structural feature for cytotoxicity against the tested cell lines [55]. It should be noted that ophiobolin O (**122**) has previously been shown to induce cell apoptosis in human breast cancer MCF-7 cells via activation of mitogen-activated protein kinase (MAPK) signaling pathways [56]. Moreover, inverse docking analysis suggested that **122** could bind to glycogen synthase kinase 3 beta (GSK3β), which is an upstream regulator of G1 phase [57]. Accordingly, it was shown that GSK3β knocked-down MCF-7 cells were not sensitive to ophiobolin O (**122**) treatment, indicating that the latter may target GSK3β to induce G1 phase arrest in MCF-7 cells. In addition, **122** treatment resulted in decreased phosphorylation levels of AKT (protein kinase B) and GSK3β, as well as in the protein expression level of cyclin D1, whereas pre-treatment with phosphatase inhibitor sodium orthovanadate blocked **122**-induced G1 phase arrest. These results indicated that the anti-proliferative effect of **122** in MCF-7 cells may be mediated through interaction with the Akt/GSK3β/cyclin D1 pathway. Besides, **122** suppressed tumorigenesis in a mouse xenograft model, whereas it showed no apparent cytotoxicity [57]. Furthermore, **122** significantly reversed adriamycin resistance in human breast cancer MCF-7/ADR cells (11-fold) at low micromolar concentrations (0.1 μM; less than 20% inhibition concentration) [57]. The reversal effect of **122** was suggested to be via elevated expression of pro-apoptotic proteins, as well as downregulation of resistance-related proteins, especially of P-glycoprotein, in MCF-7/ADR cells. Moreover, **122** enhanced mitochondrial apoptosis pathway and G2/M cell cycle arrest caused by adriamycin, due to increased level of ROS in MCF-7/ADR cells [58]. Remarkably, combination treatment of **122** and adriamycin resulted in significant tumor growth suppression (70%) in nude mice, suggesting **122** as a promising lead structure for multidrug resistance cancer chemotherapy.

#### 2.2.2. Compounds with Lipid-Lowering Activity

**Figure 18:**
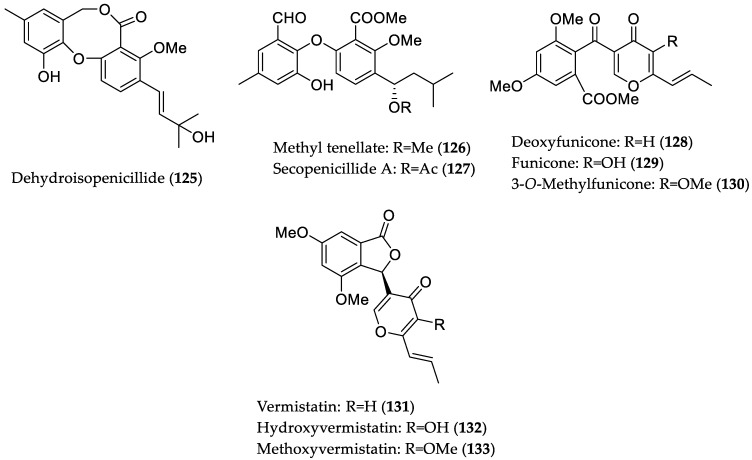
Chemical structures of **125**–**133**.

Chromatographic work-up of sediment-derived *Penicillium pinophilum* H608 (collected from the Xiamen coastline, China) extract resulted in isolation of a series of phenolic compounds, which were evaluated for their inhibitory effects against oleic acid-elicited lipid accumulation in HepG2 cells [59]. As a result of this bioactivity screening, eight compounds (**125**–**132**) (Figure 18) were found to inhibit lipid accumulation at a dose of 10 μM, with no significant cytotoxicity (IC_50_ > 50 μM). Further investigation revealed five compounds (**125**, **128**, and **130**–**132**) that significantly suppressed intracellular total cholesterol and triglycerides. Remarkably, the analogs **125**, **130**–**132** were more active than the positive control simvastatin. A real-time quantitative PCR experiment indicated that compounds **125**, **128**, and **130**–**132** affect the genes responsible for enzymes involved in lipid metabolism, including downregulation of the expression of fatty acid synthase, acetyl-CoA carboxylase, and 3-hydroxy-3-methylglutaryl-CoA reductase (inhibition of lipogenesis), as well as upregulation of carnitinepalmitoyl transferase-1 (stimulation of lipid catabolism). Moreover, metabolites **125**, **127**, **128**, and **130**–**133** reduced oxidized low-density lipoprotein stimulated lipid accumulation in RAW264.7 cells. Among the active derivatives in the latter assay, compounds **125** and **132** revealed the most pronounced effect comparable to the positive control rosiglitazone at a dose of 10 μM. Further bioassays showed that compounds **125**, **128**, and **131**–**133** significantly decreased the intracellular total cholesterol levels, although congeners **127** and **130** were inactive in RAW264.7 macrophages. Further mechanistic studies revealed that compounds **128** and **131**–**133** significantly inhibited cholesterol uptake in RAW264.7, whereas **125** and **131**–**133** stimulated cholesterol efflux to HDL. Compounds **125** and **132** showed a cholesterol efflux effect comparable to rosiglitazone and caused upregulation of mRNA levels of key regulators, such as peroxisome proliferator-activated receptor-*γ* (PPAR-*γ*), liver X receptor *α* (LXR*α*), and ATP-binding cassette G1 (ABCG1). Similarly, compounds **131** and **133** showed significant inhibition of cholesterol influx, which was slightly weaker than that of rosiglitazone, as well as stimulation of cholesterol efflux. However, both compounds did not affect transcription of the aforementioned cholesterol efflux stimulators, suggesting an unknown mechanism of the action for regulation of cholesterol efflux. Furthermore, congeners **131**–**133** decreased CD36 and SR-1 (critical scavenger receptors for regulation of cholesterol dynamics) transcription [59]. Thus, the aforementioned phenolic compounds represent new natural product leads that can be utilized for the development of hypolipidemic and anti-atherosclerotic agents (Figure 18).

### 2.3. Cytotoxic Compounds Derived from Bacteria Originating from Mangrove (Rhizosphere) Soil/Sediment Samples

**Figure 19:**
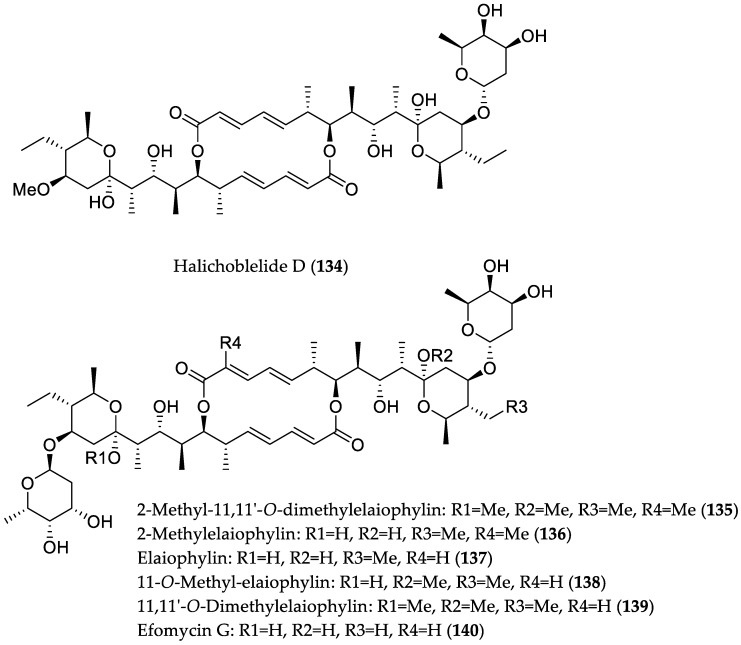
Chemical structures of **134**–**140**.

The culture broth of the actinomycete strain *Streptomyces* sp. 219807 derived from mangrove soil collected in Hainan Province, China, revealed a remarkably high yield of glycosylated 16-membered macrolide derivatives belonging to the elaiophylin family [60]. Specifically, *Streptomyces* sp. 219807 was cultured on 18 different media, and was shown to produce the highest yield of elaiophylin (up to 4486 mg/L) in shake-flasks containing DO fermentation medium. The high yield of elaiophylin was attributed to both the strain of microorganism and the DO medium containing complex carbon sources. Subsequent chemical investigation of the respective fermentation extract afforded a new elaiophylin metabolite, halichoblelide D (**134**), along with several known analogs (**135**–**140**). Compounds **134**–**140** exhibited potent cytotoxicity against HeLa and MCF-7 cell lines, with IC_50_ values in the range from 0.19 to 2.12 μM, which renders them promising lead structures for the development of anticancer agents (Figure 19).

**Figure 20:**
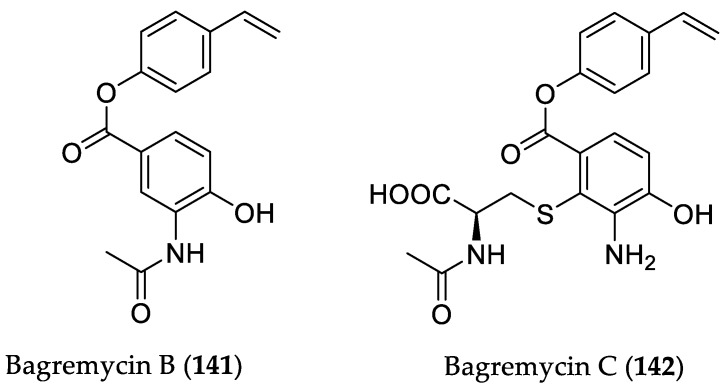
Chemical structures **141** and **142**.

Bioassay-guided investigation of the mangrove-derived actinomycete *Streptomyces* sp. Q22, isolated from a sample of mangrove soil (Guangdong, China), afforded eight natural products, including the known bagremycin B (**141**) and a new derivative bagremycin C (**142**) that showed cytotoxic properties (Figure 20) [61]. Compound **142** was found to be the most active analog against four human glioma cells, with IC_50_ values ranging between 2.2 and 6.4 μM, followed by **141**, with IC_50_ values from 7.3 to 13.3 μM, thus indicating that the *N*-acetyl-(*S*)-cysteine moiety plays an important role in the cytotoxicity of these metabolites. Interestingly, bagremycin C (**142**) at concentrations of 2.2 and 4.4 μM was found to induce late apoptosis (after 48 and 72h) in U87MG cells in a dose- and time-dependent manner. Furthermore, a U87MG cell cycle assay showed that the cell population at the G0/G1 phase was enhanced after 12 h of exposure to 4.4 μM bagremycin C (**142**), indicating that the latter might block the cell cycle at the G0/G1 phase.

**Figure 21:**
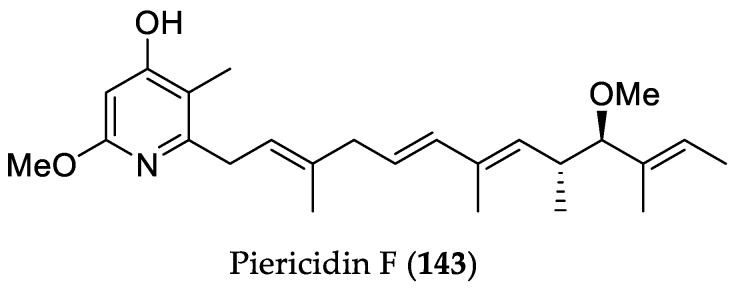
Chemical structure of **143**.

In 2013 and 2015, the *Streptomyces* sp. strain CHQ-64, obtained from rhizosphere soil collected from the mangrove conservation area of Guangdong province, China, was reported to be a source of intriguing natural products [62,63]. Chemical investigation of the crude extract of this actinomycete led to the isolation of hybrid isoprenoids (indotertines A and B, and drimentines C and H) as well as skipped-polyol polyene macrolides (reedsmycins A–F) with unprecedented scaffolds [62,63]. In a subsequent study, a mutant strain Δ*rdmF* of *Streptomyces* sp. CHQ-64 was obtained by knockout of the regulatory gene *rdmF* involved in reedsmycins biosynthesis [64]. Chemical investigation of the Δ*rdmF* mutant strain afforded an unusual 2,3,4-trisubstituted pyrrole, namely geranylpyrrol A, along with a new alkaloid piericidin F (**143**). Interestingly, the latter exhibited pronounced activity toward HeLa, NB4 (acute promyelocytic leukemia), A549, and H1975 cell lines, with IC_50_ values in the range between 0.003 to 0.56 μM, whereas geranylpyrrol A was inactive (Figure 21).

**Figure 22:**
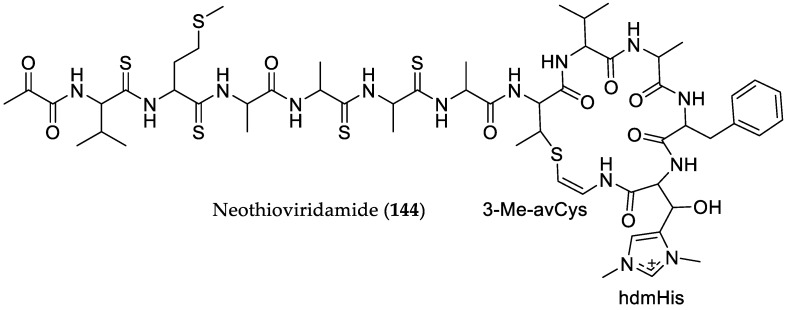
Chemical structure of **144**.

Hayakawa et al., in the course of screening for antitumor compounds using 3Y1 rat fibroblasts transformed with adenovirus oncogenes, reported a novel *N*-acylated undecapeptide, thioviridamide, from the culture broth of the actinomycete *Streptomyces olivoviridis* [65]. Remarkably, thioviridamide showed selective cytotoxicity against 3Y1 rat fibroblast cells transformed with adenovirus type 12 (Ad12-3Y1) and adenovirus E1A gene (E1A-3Y1), with IC_50_ values of 2.3 nM (3.9 ng/mL) and 23.8 nM (32 ng/mL), respectively. Significant numbers of Ad12-3Y1 cells treated with thioviridamide contained condensed chromatin and fragmented nuclei, indicating that thioviridamide induced apoptosis. In a subsequent study, the gene cluster for the biosynthesis of thioviridamide in *S. olivoriridis* NA05001 was identified and heterologously produced in *Streptomyces lividans* TK23 [66]. In addition, during genome mining for thioviridamide-like biosynthetic gene clusters, a novel cryptic biosynthetic gene cluster was identified from *Streptomyces* sp. MSB090213SC12 strain, obtained from mangrove soil in Ishigaki Island, Okinawa, Japan. In order to induce the expression of the cryptic metabolite, various fermentation media were employed, including the production medium for thioviridamide. However, this proved to be unsuccessful for the production of the novel analog. Nevertheless, heterologous expression of the respective biosynthetic gene cluster in *Streptomyces avermitilis* SUKA22 strain resulted in the production of the new derivative neothioviridamide (**144**) possessing four thioamide bonds and the unusual amino acids *β*-hydroxy-*N*^1^,*N*^3^-dimethylhistidinium (hdmHis) and 3-methyl-*S*-(2-aminovinyl)cysteine (3-Me-avCys) [67]. Interestingly, neothioviridamide (**144**) displayed cytotoxic activities against SKOV-3 (human ovarian adenocarcinoma), Meso-1 (malignant pleural mesothelioma), and Jurkat cells with IC_50_ values of 2.1, 0.7, and 0.4 μM, respectively (Figure 22).

**Figure 23:**
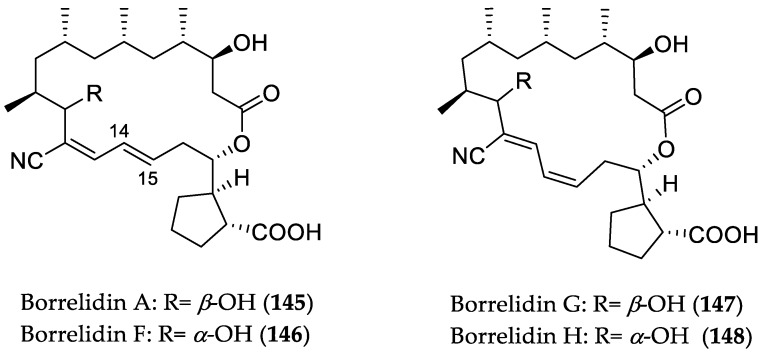
Chemical structures of **145**–**148**.

Sun et al. reported the isolation of four new macrolactone polyketide natural products of the borrelidin family, namely, borrelidin F (**146**), borrelidin G (**147**), borrelidin H (**148**), and borrelidin I, in addition to the known analogue borrelidin A (**145**) (Figure 23) [68]. These metabolites were obtained from *Streptomyces rochei* SCSIO ZJ89, which was originated from a mangrove-derived sediment sample collected in Yalongwan, China. All compounds were investigated for cytotoxicity against A549, CNE2 (nasopharyngeal carcinoma), HeLa, HepG2, and MCF-7 cell lines, as well as against normal hepatic L02 and normal umbilical vein endothelial Huvec-12 cells. Compounds **145**–**148** were active toward the respective cell lines, with IC_50_ values in the range from 0.12 to 22.75 μM. Among them, compounds **145** and 1**48** were the most active with IC_50_ values between 0.12 and 2.19 μM, stronger than those of the positive controls doxorubicin (IC_50_ = 1.02–3.52 μM) and cisplatin (IC_50_ = 2.30–12.85 μM). However, compound **148** was found to be less active toward the non-cancerous cell lines than **145**, which might be attributed to both the *α*-OH configuration and cis geometry of the C14–C15 olefin in its structure compared to the latter. Due to its selectivity toward cancer cells, **148** was further investigated on tumor cell migration, employing an in vitro wound-healing assay. Remarkably, **148** effectively inhibited tumor (HeLa and A549) cell migration, even at low micromolar concentrations (1/2 IC_50_). Moreover, it exerted little influence upon non-malignant human umbilical vein endothelial (Huvec-12) cells, which renders **148** a potential new antitumor lead compound with both cytotoxic and antimetastatic properties. Interestingly, the known analog borrelidin A (**145**) has been reported to be an allosteric inhibitor of threonyl-tRNA synthetase (ThrRS), thus preventing normal protein synthesis [69]. Moreover, its cytotoxic effect is associated with induction of G0/G1 cell cycle arrest and caspase-mediated cell death via the MAPK signaling pathway [70]. Interestingly, in a recent study, **145** was shown to increase the levels of unfolded protein response (UPR) associated with ER stress, leading to C/EBP homologous protein (CHOP)-dependent cell death in oral squamous cell carcinoma cells [71]. Therefore, **148** might exert similar mechanisms for selectively targeting cancer cells.

**Figure 24:**
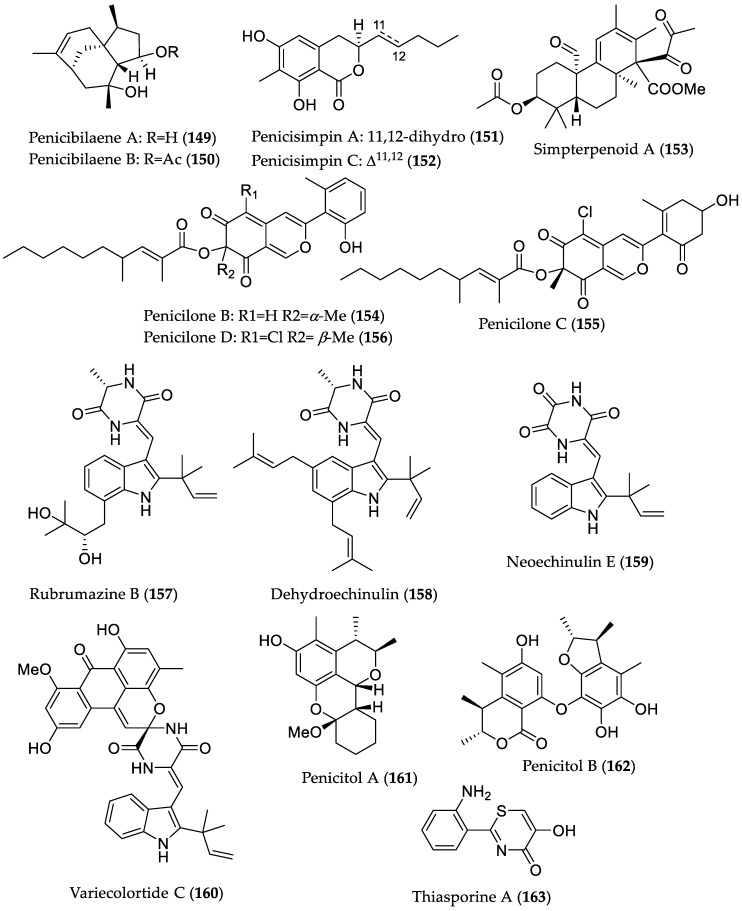
Chemical structures of bioactive compounds from mangrove fungi and bacteria derived from soil/sediment samples (**149**–**163**).

Further bioactive metabolites derived from mangrove soil/sediment microorganisms that could serve as potential lead structures are summarized in Table 2 and Figure 24.

## 3. Conclusions and Outlook

Mangrove-associated microorganisms have gained considerable attention as a rich source of structurally diverse secondary metabolites with pronounced biological activities, which could be utilized in the discovery of new drug leads [79]. In this review, 163 compounds have been presented from mangrove-associated microorganisms, the majority of which show remarkable activities, such as the potent cytotoxic penicisulfuranols A–C (**100**–**102**) [48], phomoxanthone A (**19**) [32], and piericidin F (**143**) [64], as well as the anti-inflammatory rhytidenones C and D (**40** and **41**) [34]. Nevertheless, these metabolites represent only a small fraction of the biosynthetic capacity of the source microorganisms, as predicted through genomic studies [80]. This is partially due to the fact that most of the biosynthetic gene clusters expressing novel bioactive metabolites remain silent (or cryptic) under standard laboratory culture conditions, and thus the metabolic potential of these microorganisms remains untapped [81,82]. Taking this fact into account, new methods and technologies are warranted to activate cryptic pathways and explore the secondary metabolome of microbes [83].

The biosynthetic potential of mangrove-associated microorganisms has been associated with activation of silent genes by unique environmental stimuli imparted on this special ecological niche [84]. Thus, production of cryptic metabolites may be accomplished by altering culture conditions, such as temperature, media, pH, and light, or by adding elicitors/chemicals, e.g., DMSO, inorganic salts, and plant exudates—in the case of endophytes—to the culture [79,82]. Alteration of culture conditions, the so-called OSMAC approach, has been successfully exploited to generate novel compounds from mangrove-associated fungi, such as brocapyrrozin A (**25**) and lasiodiplactone A (**42**) from the endophytic fungi *P. brocae* MA-231 [26] and *L. theobromae* ZJ-HQ1 [35], respectively.

Co-cultivation of microorganisms has likewise been exploited to enhance the accumulation of constitutively present natural products and/or to trigger the production of cryptic metabolites [85], which could not be achieved though fermentation of individual strains. It is assumed that microbial interactions imitate the natural habitat of microbes, either in a symbiotic relationship or in competition for nutrients and space, and thus play an important role in the activation of silent genes [84]. This methodology has led to the induction of several novel cryptic metabolites, including citrifelins A (**68**) and B (**69**) from co-culture of the mangrove endophyte *P. citrinum* MA-197 with the bryozoan-derived *B. felina* EN-135 [42], as well as microsphaeropsisin C (**75**) and lasiodiplodin derivatives (**76**–**79**) with *α*-glucosidase inhibitory activity from co-culture of the endophytic fungus *Trichoderma* sp. 307 with *A. johnsonii* B2 [44].

In the last few years, epigenetic manipulation has attracted great interest as a strategy for activation of silent biosynthetic pathways. Several regulatory proteins, such as chromatin-modulating agents and transcription factors, are known to control the secondary metabolome of microbes [84]. The epigenetic modifiers suberoylanilide hydroxamic acid (SAHA) and 5-azacytidine that inhibit the activities of histone deacetylases and DNA methyltransferases, respectively, have proven to be effective in activation of silenced gene clusters [83,84]. For instance, addition of 5-azacytidine to the mangrove-associated fungus *P. variabile* HXQ-H-1 afforded the cryptic metabolite varitatin A (**113**) with potent cytotoxic and protein kinase inhibitory activities [52]. Notably, it has been shown that secondary metabolite production in *Aspergillus nidulans* was triggered by co-cultivation with the bacterium *Streptomyces rapamycinicus* due to targeted histone modification, thus shedding light on the connection between epigenetic modification and microbial crosstalk [86]. 

The majority of compounds described in this review have been derived from common genera, such as *Streptomyces* and *Penicillium*. Even though these microorganisms still hold enormous biosynthetic potential, as demonstrated by recent genome sequence studies, a vast amount of novel fungal and/or bacterial taxonomic groups still lies unexplored [79]. To a large extent, this is due to limitations in traditional isolation procedures, as well as the non-culturable feature of many of these latter microorganisms [87]. To overcome this problem, molecular approaches, such as high-throughput sequencing and metagenomics, have become promising tools for unraveling the biosynthetic potential of hitherto uncultured microorganisms, evading the need for isolation of individual species [87,88], and thus providing an inexhaustible source of new microbial taxa. Genome mining and bioinformatics approaches also serve as powerful strategies towards the prediction of key biosynthetic clusters from genome sequence analysis data, providing a wealth of information that can be linked to cryptic secondary metabolites, as exemplified in the case of neothioviridamide (**144**) from *Streptomyces* sp. MSB090213SC12 [67,89,90]. These novel biosynthetic clusters can be genetically engineered and expressed in heterologous hosts, such as *Saccharomyces cerevisiae*, *E. coli*, or *Streptomyces lividans* for large-scale production of the desired lead compounds or derivatives thereof [66,91,92].

Overall, mangrove-associated microorganisms have gained considerable attention due to their unique ecological characteristics, diversity, and wealth of novel bioactive secondary metabolites. Nevertheless, pharmaceutical development of these metabolites is still in its infancy, with only the proteasome inhibitor, salinosporamide A, being hitherto in phase I clinical trials for the treatment of multiple myeloma [12,93]. In order to unravel the metabolic potential of mangrove endophytes, an integrative understanding of the principal molecular mechanisms involved in the regulation of natural product biogenesis is essential [88]. Strategies to activate silent genes for exploration of novel compounds, such as epigenetic modification, OSMAC, and microbial co-cultivation approaches, along with continuing advancements in modern “omics” methodologies, including transcriptomics, proteomics, and metabolomics, is expected to open up an exciting area of research for the discovery of lead compounds from mangrove-associated microorganisms in the coming years.

## Figures and Tables

**Table 1:** Further bioactive compounds isolated from endophytic fungi (**63**–**105**) of mangrove origin.

Compound Name	Source	Type of Activity [Ref.]	IC_50_ or MIC Values
Peniphenone B (**63**)	*Penicillium dipodomyicola* HN4-3A from *Acanthus ilicifolius* (Hainan Province, China)	MptpB inhibitory [39]	IC_50_ 0.16 μM
Peniphenone C (**64**)			IC_50_ 1.37 μM
Talaramide A (**65**)	*Talaromyces* sp. HZ-YX1 from *Kandelia obovata* (Guangdong Province, China)	Mycobacterial serine/threonine protein kinase G (PknG) inhibitory [40]	IC_50_ 55 μM, positive control AX20017 (IC_50_ = 98 μM) ^1^
Neosartoryadin A (**66**)	*Neosartorya udagawae* HDN13-313 from *Avicennia marina* (Hainan Province, China)	Anti-influenza A virus (H1N1) [41]	IC_50_ 66 μM; positive control ribavirin (IC_50_ 94 μM); no cytotoxicity against the human leukemia (HL-60) cell line
Neosartoryadin B (**67**)			IC_50_ 58 μM; -//-
Citrifelin A (**68**)	Co-culture of *Penicillium citrinum* MA-197 (from *Lumnitzera racemosa*) and *Beauveria felina* EN-135	Antibacterial against *E. coli* and *S. aureus* [42]	MIC 24.5 μM/8.0 μg/mL (both *E. coli* and *S. aureus*)
Citrifelin B (**69**)			MICs 5.6 μM/2.0 μg/mL (*E. coli*) and 11.2 μM/4.0 μg/mL (*S. aureus*)
Pinazaphilone A (**70**)	*Penicillium* sp. HN29-3B1 from *Cerbera manghas* (Hainan Island, China)	*α*-Glucosidase inhibitory [43]	IC_50_ 81.7 μM; positive control acarbose (IC_50_ = 446.7 μM)
Pinazaphilone B (**71**)			IC_50_ 28.0 μM; -//-
6′-Methyl-[1,1′-biphenyl]-3,3′,4′,5-tetraol (**72**)			IC_50_ 2.2 μM; -//-
Sch 1385568 (**73**)			IC_50_ 16.6 μM; -//-
(±)-Penifupyrone (**74**)			IC_50_ 14.4 μM; -//-
Microsphaeropsisin C (**75**)	Co-culture of *Trichoderma* sp. 307 (from *Clerodendrum inerme*; Guangdong Province, China) with *Acinetobacter johnsonii* B2	*α*-Glucosidase inhibitory [44]	IC_50_ 188.7 μM; positive control acarbose (IC_50_ = 703.8 μM)
(3*R*,7*R*)-7-Hydroxy-de-*O*-methyllasiodiplodin (**76**)			IC_50_ 25.8 μM; -//-
(3*R*)-5-Oxo-de-*O*-methyllasiodiplodin (**77**)			IC_50_ 54.6 μM; -//-
(3*R*)-7-Oxo-de-*O*-methyllasiodiplodin (**78**)			IC_50_ 178.5 μM; -//-
(3*R*)-5-Oxolasiodiplodin (**79**)			IC_50_ 176.8 μM; -//-
(3*R*,4*R*)-4-Hydroxy-de-*O*-methyllasiodiplodin (**80**)			IC_50_ 60.3 μM; -//-
(3*S*)-Ozoroalide (**81**)			IC_50_ 198.1 μM; -//-
(*E*)-9-Etheno-lasiodiplodin (**82**)			IC_50_ 101.3 μM; -//-
Secalonic acid A (**83**)	Plant endophyte *Setophoma terrestris* from a leaf litter collected in a mangrove habitat	Cytotoxic against MDA-MB-435 (melanoma) and SW-620 (colon cancer) cell lines [45]	IC_50_ 0.16 (MDA-MB-435) and 0.41 μM (SW-620)
Penicillixanthone A (**84**)			IC_50_ 0.18 (MDA-MB-435) and 0.21 μM (SW-620)
Blennolide J (**85**)			IC_50_ 4.06 (MDA-MB-435) and 6.14 μM (SW-620)
Hypothemycin (**86**)			IC_50_ 0.58 (MDA-MB-435) and 2.14 μM (SW-620)
Penicillixanthone B (**87**)			IC_50_ 5.20 (MDA-MB-435) and 5.55 μM (SW-620)
Secalonic acid G (**88**)		Cytotoxic against MDA-MB-435 and SW-620 cell lines/antibacterial against *M. luteus* [45]	IC_50_ 3.27 (MDA-MB-435) and 3.67 μM (SW-620)/MIC 7.83 μM (/5 μg/mL)
Rhizovarin A (**89**)	*Mucor irregularis* QEN-189 from *Rhizophora stylosa* (Hainan Island, China)	Cytotoxic against A549 and/or HL-60 (promyelocytic leukemia) cancer cell lines [46]	9.6 μM (HL-60)
Rhizovarin B (**90**)			6.3 (A549) and 5.0 μM (HL-60)
Rhizovarin E (**91**)			9.2 μM (A549)
Penitrem A (**92**)			8.4 (A549) and 7.0 μM (HL-60)
Penitrem C (**93**)			8.0 (A549) and 4.7 μM (HL-60)
Penitrem F (**94**)			8.2 (A549) and 3.3 μM (HL-60)
10*β*-Hydroxy-13-desoxypaxilline (**95**)			4.6 (A549) and 2.6 μM (HL-60)
7-*O*-Methylnigrosporolide (**96**)	*Pestalotiopsis microspora* from *Drepanocarpus lunatus* (Cameroon)	Cytotoxic against L5178Y (murine lymphoma) cell line or human ovarian (A2780) cancer cell line [47]	IC_50_ 0.7 μM (L5178Y)
Pestalotioprolide D (**97**)			IC_50_ 5.6 μM (L5178Y)
Pestalotioprolide E (**98**)			IC_50_ 3.4 (L5178Y) and 1.2 μM; (A2780)
Pestalotioprolide F (**99**)			IC_50_ 3.9 μM (L5178Y)
Penicisulfuranol A (**100**)	*Penicillium janthinellum* HDN13-309 from *Sonneratia caseolaris* (Hainan Province, China)	Cytotoxic against HeLa and HL-60 cell lines [48]	IC_50_ 0.5 (HeLa) and 0.1 (HL-60) μM;
Penicisulfuranol B (**101**)			IC_50_ 3.9 (HeLa) and 1.6 μM (HL-60)
Penicisulfuranol C (**102**)			IC_50_ 0.3 (HeLa) and 1.2 μM (HL-60)
Penicilindole A (**103**)	*Eupenicillium* sp. HJ002 from *Xylocarpus granatum* (South China Sea)	Cytotoxic against A549 and HepG2 cell lines [49]	IC_50_ 5.5 (A549) and 1.5 (HepG2) μM
*epi*-Isochromophilone II (**104**)	*Diaporthe* sp. SCSIO 41011 from *Rhizophora stylosa* (Hainan Province, China)	Cytotoxic against renal carcinoma cell lines: ACHN, OS-RC-2, and 786-O [50]	IC_50_ 4.4 (ACHN), 3.0 (786-O) and 3.9 μM (OS-RC-2)
Isochromophilone D (**105**)			IC_50_ 14 (ACHN), 8.9 (786-O) and 13 μM (OS-RC-2); induced apoptosis (in 786-O cells) in a dose- and time-dependent manner, whereas it did not induce cell cycle arrest at a concentration level up to 10 μM.

^1^ Positive control is indicated in case the IC_50_ value of the respective compound is higher than 10 μM.

**Table 2:** Bioactive compounds isolated from soil-derived fungi (**149**–**162**) and bacteria (**163**) of mangrove origin.

Penicibilaene A (**149**)	*Penicillium bilaiae* MA-267 from the rhizospheric soil of *Lumnitzera racemosa* (Hainan Island, China)	Antifungal against *Colletotrichum gloeosporioides* [72]	MIC 4.23 μM/1.0 μg/mL
Penicibilaene B (**150**)			MIC 0.45 μM/0.125 μg/mL
Penicisimpin A (**151**)	*Penicillium simplicissimum* MA-332 from the rhizospheric soil of *B. sexangula var. rhynchopetala* (Hainan Island, China)	Antibacterial and antifungal [73]	MIC 15.1 μM/4.0 μg/mL (*E. coli*, *P. aeruginosa*, *Vibrio harveyi*, *Vibrio parahaemolyticus C. gloeosporioides*) and 30.3 μM (8 μg/mL) (*M. luteus*, *Vibrio alginolyticus*) MIC 15.1 μM/4.0 μg/mL (*C. gloeosporioides*)
Penicisimpin C (**152**)			MIC 30.5 μM/8.0 μg/mL (*E. coli*, *P. aeruginosa*, *V. harveyi*, *V. parahaemolyticus*) MIC 30.5 μM (8.0 μg/mL) (*C. gloeosporioides*)
Simpterpenoid A (**153**)		Influenza neuraminidase inhibitory activity [74]	IC_50_ 8.1 nM
Penicilone B (**154**)	*Penicillium janthinellum* HK1-6, isolated from mangrove rhizosphere soil (Dongzhaigang, Hainan Island)	Antibacterial against methicillin-resistant and -susceptible *S. aureus*, vancomycin-resistant *Enterococcus faecalis*, and -susceptible *Enterococcus faecium* strains [75]	MIC 6.54 μM/3.13 μg/mL
Penicilone C (**155**)			MIC 11.8–23.5 μM (6.25–12.5 μg/mL)
Penicilone D (**156**)			MIC 6.1–24.4 μM (3.13–12.5 μg/mL)
Rubrumazine B (**157**)	*Eurotium rubrum* MA-150 from mangrove-derived rhizospheric soil (Andaman Sea coastline, Thailand)	Cytotoxic in brine shrimp assay [76]	LD_50_ 2.4 μM
Dehydroechinulin (**158**)			LD_50_ 3.5 μM
Neoechinulin E (**159**)			LD_50_ 3.9 μM
Variecolortide C (**160**)			LD_50_ 9.8 μM
Penicitol A (**161**)	*Penicillium chrysogenum* HND 11–24 from the rhizosphere soil of *Acanthus ilicifolius*	Cytotoxic against several cancer cell lines and HEK 293 [77]	IC_50_ 4.6–7.6 μM; HeLa, HEK 293, HCT-116, and A549 cell lines
Penicitol B (**162**)			IC_50_ 3.4–9.6 μM; HeLa, BEL-7402 (hepatocellular carcinoma), HEK 293, HCT-116, and A549 cell lines
Thiasporine A (**163**)	*Actinomycetospora chlora* SNC-032 from mangrove swamp sediment sample (Vava’u, Tonga)	Cytotoxic toward non-small-cell lung cancer H2122 cell line [78]	IC_50_ 5.4 μM

## References

[1] Tomlinson P.B. (2016). Ecology. The Botany of Mangroves.

[2] Wu J., Xiao Q., Xu J., Li M.-Y., Pan J.-Y., Yang M.-H. (2008). Natural products from true mangrove flora: Source, chemistry and bioactivities. Nat. Prod. Rep..

[3] Saenger P. (2002). Mangrove Ecology, Silviculture and Conservation.

[4] Wang L., Mu M., Li X., Lin P., Wang W. (2011). Differentiation between true mangroves and mangrove associates based on leaf traits and salt contents. J. Plant Ecol..

[5] Yin S., Fan C.-Q., Wang X.-N., Lin L.-P., Ding J., Yue J.-M. (2006). Xylogranatins A−D:  Novel tetranortriterpenoids with an unusual 9,10-*seco* scaffold from marine mangrove *Xylocarpus granatum*. Org. Lett..

[6] Li W.S., Wu J., Li J., Satyanandamurty T., Shen L., Bringmann G. (2017). Krishnadimer A, an axially chiral non-biaryl natural product: Discovery and biomimetic synthesis. Org. Lett..

[7] Gong K.-K., Li P.-L., Qiao D., Zhang X.-W., Chu M.-J., Qin G.-F., Tang X.-L., Li G.-Q. (2017). Cytotoxic and antiviral triterpenoids from the mangrove plant *Sonneratia paracaseolaris*. Molecules.

[8] Zhang Q., Satyanandamurty T., Shen L., Wu J. (2017). Krishnolides A–D: New 2-ketokhayanolides from the Krishna mangrove, *Xylocarpus moluccensis*. Mar. Drugs.

[9] Li J., Li M.-Y., Bruhn T., Katele F.Z., Xiao Q., Pedpradab P., Wu J., Bringmann G. (2013). Thaixylomolins A–C: Limonoids featuring two new motifs from the Thai *Xylocarpus moluccensis*. Org. Lett..

[10] Fei Y., Li X.-W., Guo Y.-W. (2016). Recent progress on the mangrove plants: Chemistry and bioactivity. Curr. Org. Chem..

[11] Lin Y., Wu X., Feng S., Jiang G., Luo J., Zhou S., Vrijmoed L.L.P., Jones E.B.G., Krohn K., Steingröver K. (2001). Five unique compounds:  Xyloketals from mangrove fungus *Xylaria* sp. from the South China Sea coast. J. Org. Chem..

[12] Feling R.H., Buchanan G.O., Mincer T.J., Kauffman C.A., Jensen P.R., Fenical W. (2003). Salinosporamide A: A highly cytotoxic proteasome inhibitor from a novel microbial source, a marine bacterium of the new genus *Salinospora*. Angew. Chem. Int. Ed..

[13] MarinLit. http://pubs.rsc.org/marinlit.

[14] Blunt J.W., Carroll A.R., Copp B.R., Davis R.A., Keyzers R.A., Prinsep M.R. (2018). Marine natural products. Nat. Prod. Rep..

[15] Simões M.F., Antunes A., Ottoni C.A., Amini M.S., Alam I., Alzubaidy H., Mokhtar N.-A., Archer J.A.C., Bajic V.B. (2015). Soil and rhizosphere associated fungi in gray mangroves (*Avicennia marina*) from the Red Sea—A metagenomic approach. Genom. Proteom. Bioinform..

[16] Sanka Loganathachetti D., Poosakkannu A., Muthuraman S. (2017). Fungal community assemblage of different soil compartments in mangrove ecosystem. Sci. Rep..

[17] Mendes L., Tsai S. (2014). Variations of bacterial community structure and composition in mangrove sediment at different depths in Southeastern Brazil. Diversity.

[18] Basak P., Pramanik A., Sengupta S., Nag S., Bhattacharyya A., Roy D., Pattanayak R., Ghosh A., Chattopadhyay D., Bhattacharyya M. (2016). Bacterial diversity assessment of pristine mangrove microbial community from Dhulibhashani, Sundarbans using 16S rRNA gene tag sequencing. Genom. Data.

[19] Liang J.-B., Chen Y.-Q., Lan C.-Y., Tam N.F.Y., Zan Q.-J., Huang L.-N. (2007). Recovery of novel bacterial diversity from mangrove sediment. Mar. Biol..

[20] De Souza Sebastianes F.L., Romão-Dumaresq A.S., Lacava P.T., Harakava R., Azevedo J.L., de Melo I.S., Pizzirani-Kleiner A.A. (2013). Species diversity of culturable endophytic fungi from Brazilian mangrove forests. Curr. Genet..

[21] Xu D.-B., Ye W.-W., Han Y., Deng Z.-X., Hong K. (2014). Natural products from mangrove actinomycetes. Mar. Drugs.

[22] Xu J. (2015). Bioactive natural products derived from mangrove-associated microbes. RSC Adv..

[23] Meng L.-H., Li X.-M., Lv C.-T., Huang C.-G., Wang B.-G. (2014). Brocazines A–F, cytotoxic bisthiodiketopiperazine derivatives from *Penicillium brocae* MA-231, an endophytic fungus derived from the marine mangrove plant *Avicennia marina*. J. Nat. Prod..

[24] Meng L.-H., Wang C.-Y., Mándi A., Li X.-M., Hu X.-Y., Kassack M.U., Kurtán T., Wang B.-G. (2016). Three diketopiperazine alkaloids with spirocyclic skeletons and one bisthiodiketopiperazine derivative from the mangrove-derived endophytic fungus *Penicillium brocae* MA-231. Org. Lett..

[25] Meng L.-H., Zhang P., Li X.-M., Wang B.-G. (2015). Penicibrocazines A–E, five new sulfide diketopiperazines from the marine-derived endophytic fungus *Penicillium brocae*. Mar. Drugs.

[26] Meng L.-H., Li X.-M., Liu Y., Xu G.-M., Wang B.-G. (2017). Antimicrobial alkaloids produced by the mangrove endophyte *Penicillium brocae* MA-231 using the OSMAC approach. RSC Adv..

[27] Chen S., Chen D., Cai R., Cui H., Long Y., Lu Y., Li C., She Z. (2016). Cytotoxic and antibacterial preussomerins from the mangrove endophytic fungus *Lasiodiplodia theobromae* ZJ-HQ1. J. Nat. Prod..

[28] Liu Y., Stuhldreier F., Kurtan T., Mandi A., Arumugam S., Lin W., Stork B., Wesselborg S., Weber H., Henrich B. (2017). Daldinone derivatives from the mangrove-derived endophytic fungus *Annulohypoxylon* sp.. RSC Adv..

[29] Siridechakorn I., Yue Z., Mittraphab Y., Lei X., Pudhom K. (2017). Identification of spirobisnaphthalene derivatives with anti-tumor activities from the endophytic fungus *Rhytidhysteron rufulum* AS21B. Bioorg. Med. Chem..

[30] Marian F., Hendrik N., Philip B., Björn S., Sebastian W., Wenhan L., Peter P. (2015). Phomoxanthone A—from mangrove forests to anticancer therapy. Curr. Med. Chem..

[31] Rönsberg D., Debbab A., Mándi A., Vasylyeva V., Böhler P., Stork B., Engelke L., Hamacher A., Sawadogo R., Diederich M. (2013). Pro-apoptotic and immunostimulatory tetrahydroxanthone dimers from the endophytic fungus *Phomopsis longicolla*. J. Org. Chem..

[32] Böhler P., Stuhldreier F., Anand R., Kondadi A.K., Schlutermann D., Berleth N., Deitersen J., Wallot-Hieke N., Wu W., Frank M. (2018). The mycotoxin phomoxanthone A disturbs the form and function of the inner mitochondrial membrane. Cell Death Dis..

[33] Zhou X.-M., Zheng C.-J., Chen G.-Y., Song X.-P., Han C.-R., Li G.-N., Fu Y.-H., Chen W.-H., Niu Z.-G. (2014). Bioactive anthraquinone derivatives from the mangrove-derived fungus *Stemphylium* sp. 33231. J. Nat. Prod..

[34] Pudhom K., Teerawatananond T. (2014). Rhytidenones A–F, spirobisnaphthalenes from *Rhytidhysteron* sp. AS21B, an endophytic fungus. J. Nat. Prod..

[35] Chen S., Liu Z., Liu H., Long Y., Chen D., Lu Y., She Z. (2017). Lasiodiplactone A, a novel lactone from the mangrove endophytic fungus *Lasiodiplodia theobromae* ZJ-HQ1. Org. Biomol. Chem..

[36] Liu Y., Chen S., Liu Z., Lu Y., Xia G., Liu H., He L., She Z. (2015). Bioactive metabolites from mangrove endophytic fungus *Aspergillus* sp. 16–5B. Mar. Drugs.

[37] Chen S., Liu Y., Liu Z., Cai R., Lu Y., Huang X., She Z. (2016). Isocoumarins and benzofurans from the mangrove endophytic fungus *Talaromyces amestolkiae* possess *α*-glucosidase inhibitory and antibacterial activities. RSC Adv..

[38] Cui H., Lin Y., Luo M., Lu Y., Huang X., She Z. (2017). Diaporisoindoles A–C: Three isoprenylisoindole alkaloid derivatives from the mangrove endophytic fungus *Diaporthe* sp. SYSU-HQ3. Org. Lett..

[39] Li H., Jiang J., Liu Z., Lin S., Xia G., Xia X., Ding B., He L., Lu Y., She Z. (2014). Peniphenones A–D from the mangrove fungus *Penicillium dipodomyicola* HN4-3A as inhibitors of *Mycobacterium tuberculosis* phosphatase MptpB. J. Nat. Prod..

[40] Chen S., He L., Chen D., Cai R., Long Y., Lu Y., She Z. (2017). Talaramide A, an unusual alkaloid from the mangrove endophytic fungus *Talaromyces* sp. (HZ-YX1) as an inhibitor of mycobacterial PknG. New J. Chem..

[41] Yu G., Zhou G., Zhu M., Wang W., Zhu T., Gu Q., Li D. (2016). Neosartoryadins A and B, fumiquinazoline alkaloids from a mangrove-derived fungus *Neosartorya udagawae* HDN13-313. Org. Lett..

[42] Meng L.-H., Liu Y., Li X.-M., Xu G.-M., Ji N.-Y., Wang B.-G. (2015). Citrifelins A and B, citrinin adducts with a tetracyclic framework from cocultures of marine-derived isolates of *Penicillium citrinum* and *Beauveria felina*. J. Nat. Prod..

[43] Liu Y., Yang Q., Xia G., Huang H., Li H., Ma L., Lu Y., He L., Xia X., She Z. (2015). Polyketides with *α*-glucosidase inhibitory activity from a mangrove endophytic fungus, *Penicillium* sp. HN29-3B1. J. Nat. Prod..

[44] Zhang L., Niaz S., Khan D., Wang Z., Zhu Y., Zhou H., Lin Y., Li J., Liu L. (2017). Induction of diverse bioactive secondary metabolites from the mangrove endophytic fungus *Trichoderma* sp. (strain 307) by co-cultivation with *Acinetobacter johnsonii* (strain B2). Mar. Drugs.

[45] El-Elimat T., Figueroa M., Raja H.A., Graf T.N., Swanson S.M., Falkinham J.O., Wani M.C., Pearce C.J., Oberlies N.H. (2015). Biosynthetically distinct cytotoxic polyketides from *Setophoma terrestris*. Eur. J. Org. Chem..

[46] Gao S.-S., Li X.-M., Williams K., Proksch P., Ji N.-Y., Wang B.-G. (2016). Rhizovarins A–F, indole-diterpenes from the mangrove-derived endophytic fungus *Mucor irregularis* QEN-189. J. Nat. Prod..

[47] Liu S., Dai H., Makhloufi G., Heering C., Janiak C., Hartmann R., Mándi A., Kurtán T., Müller W.E.G., Kassack M.U. (2016). Cytotoxic 14-membered macrolides from a mangrove-derived endophytic fungus, *Pestalotiopsis microspora*. J. Nat. Prod..

[48] Zhu M., Zhang X., Feng H., Dai J., Li J., Che Q., Gu Q., Zhu T., Li D. (2017). Penicisulfuranols A–F, alkaloids from the mangrove endophytic fungus *Penicillium janthinellum* HDN13-309. J. Nat. Prod..

[49] Zheng C.-J., Bai M., Zhou X.-M., Huang G.-L., Shao T.-M., Luo Y.-P., Niu Z.-G., Niu Y.-Y., Chen G.-Y., Han C.-R. (2018). Cytotoxic indole diterpenes from the mangrove-derived fungus *Eupenicillium* sp. HJ002. J. Nat. Prod..

[50] Luo X., Lin X., Tao H., Wang J., Li J., Yang B., Zhou X., Liu Y. (2018). Isochromophilones A–F, cytotoxic chloroazaphilones from the marine mangrove endophytic fungus *Diaporthe* sp. SCSIO 41011. J. Nat. Prod..

[51] Wu G., Yu G., Kurtán T., Mándi A., Peng J., Mo X., Liu M., Li H., Sun X., Li J. (2015). Cytotoxic xanthone–chromanone dimers from the marine-derived fungus *Aspergillus versicolor* HDN1009. J. Nat. Prod..

[52] He X., Zhang Z., Chen Y., Che Q., Zhu T., Gu Q., Li D. (2015). Varitatin A, a highly modified fatty acid amide from *Penicillium variabile* cultured with a DNA methyltransferase inhibitor. J. Nat. Prod..

[53] Zhang Z., He X., Zhang G., Che Q., Zhu T., Gu Q., Li D. (2017). Inducing secondary metabolite production by combined culture of *Talaromyces aculeatus* and *Penicillium variabile*. J. Nat. Prod..

[54] Lu Z., Wang Y., Miao C., Liu P., Hong K., Zhu W. (2009). Sesquiterpenoids and benzofuranoids from the marine-derived fungus *Aspergillus ustus* 094102. J. Nat. Prod..

[55] Zhu T., Lu Z., Fan J., Wang L., Zhu G., Wang Y., Li X., Hong K., Piyachaturawat P., Chairoungdua A. (2018). Ophiobolins from the mangrove fungus *Aspergillus ustus*. J. Nat. Prod..

[56] Yang T., Lu Z., Meng L., Wei S., Hong K., Zhu W., Huang C. (2012). The novel agent ophiobolin O induces apoptosis and cell cycle arrest of MCF-7 cells through activation of MAPK signaling pathways. Bioorg. Med. Chem. Lett..

[57] Lv C., Qin W., Zhu T., Wei S., Hong K., Zhu W., Chen R., Huang C. (2015). Ophiobolin O isolated from *Aspergillus ustus* induces G1 arrest of MCF-7 cells through interaction with AKT/GSK3β/Cyclin D1 signaling. Mar. Drugs.

[58] Sun W., Lv C., Zhu T., Yang X., Wei S., Sun J., Hong K., Zhu W., Huang C. (2013). Ophiobolin O reverses adriamycin resistance via cell cycle arrest and apoptosis sensitization in adriamycin-resistant human breast carcinoma (MCF-7/ADR) cells. Mar. Drugs.

[59] Wu C., Zhao Y., Chen R., Liu D., Liu M., Proksch P., Guo P., Lin W. (2016). Phenolic metabolites from mangrove-associated *Penicillium pinophilum* fungus with lipid-lowering effects. RSC Adv..

[60] Han Y., Tian E., Xu D., Ma M., Deng Z., Hong K. (2016). Halichoblelide D, a new elaiophylin derivative with potent cytotoxic activity from mangrove-derived *Streptomyces* sp. 219807. Molecules.

[61] Chen L., Chai W., Wang W., Song T., Lian X.-Y., Zhang Z. (2017). Cytotoxic bagremycins from mangrove-derived *Streptomyces* sp. Q22. J. Nat. Prod..

[62] Che Q., Zhu T., Keyzers R.A., Liu X., Li J., Gu Q., Li D. (2013). Polycyclic hybrid isoprenoids from a reed rhizosphere soil derived *Streptomyces* sp. CHQ-64. J. Nat. Prod..

[63] Che Q., Li T., Liu X., Yao T., Li J., Gu Q., Li D., Li W., Zhu T. (2015). Genome scanning inspired isolation of reedsmycins A–F, polyene-polyol macrolides from *Streptomyces* sp. CHQ-64. RSC Adv..

[64] Han X., Liu Z., Zhang Z., Zhang X., Zhu T., Gu Q., Li W., Che Q., Li D. (2017). Geranylpyrrol A and piericidin F from *Streptomyces* sp. CHQ-64 ΔrdmF. J. Nat. Prod..

[65] Hayakawa Y., Sasaki K., Nagai K., Shin-ya K., Furihata K. (2006). Structure of thioviridamide, a novel apoptosis inducer from *Streptomyces olivoviridis*. J. Antibiot..

[66] Izawa M., Kawasaki T., Hayakawa Y. (2013). Cloning and heterologous expression of the thioviridamide biosynthesis gene cluster from *Streptomyces olivoviridis*. Appl. Environ. Microbiol..

[67] Kawahara T., Izumikawa M., Kozone I., Hashimoto J., Kagaya N., Koiwai H., Komatsu M., Fujie M., Sato N., Ikeda H. (2018). Neothioviridamide, a polythioamide compound produced by heterologous expression of a *Streptomyces* sp. Cryptic RiPP biosynthetic gene cluster. J. Nat. Prod..

[68] Sun J., Shao J., Sun C., Song Y., Li Q., Lu L., Hu Y., Gui C., Zhang H., Ju J. (2018). Cytotoxic and cell migration inhibiting agents from mangrove-derived *Streptomyces rochei* SCSIO ZJ89. Bioorg. Med. Chem..

[69] Ruan B., Bovee M.L., Sacher M., Stathopoulos C., Poralla K., Francklyn C.S., Söll D. (2005). A unique hydrophobic cluster near the active site contributes to differences in borrelidin inhibition among threonyl-tRNA synthetases. J. Biol. Chem..

[70] Gao X., Jiang Y., Han L., Chen X., Hu C., Su H., Mu Y., Guan P., Huang X. (2017). Effect of borrelidin on hepatocellular carcinoma cells in vitro and in vivo. RSC Adv..

[71] Sidhu A., Miller J.R., Tripathi A., Garshott D.M., Brownell A.L., Chiego D.J., Arevang C., Zeng Q., Jackson L.C., Bechler S.A. (2015). Borrelidin induces the unfolded protein response in oral cancer cells and chop-dependent apoptosis. ACS Med. Chem. Lett..

[72] Meng L.-H., Li X.-M., Liu Y., Wang B.-G. (2014). Penicibilaenes A and B, sesquiterpenes with a tricyclo[6.3.1.0^1,5^]dodecane skeleton from the marine isolate of *Penicillium bilaiae* MA-267. Org. Lett..

[73] Xu R., Li X.-M., Wang B.-G. (2016). Penicisimpins A–C, three new dihydroisocoumarins from *Penicillium simplicissimum* MA-332, a marine fungus derived from the rhizosphere of the mangrove plant *Bruguiera sexangula* var. rhynchopetala. Phytochem. Lett..

[74] Li H.-L., Xu R., Li X.-M., Yang S.-Q., Meng L.-H., Wang B.-G. (2018). Simpterpenoid A, a meroterpenoid with a highly functionalized cyclohexadiene moiety featuring *gem*-propane-1,2-dione and methylformate groups, from the mangrove-derived *Penicillium simplicissimum* MA-332. Org. Lett..

[75] Chen M., Shen N.-X., Chen Z.-Q., Zhang F.-M., Chen Y. (2017). Penicilones A–D, anti-MRSA azaphilones from the marine-derived fungus *Penicillium janthinellum* HK1-6. J. Nat. Prod..

[76] Meng L.-H., Du F.-Y., Li X.-M., Pedpradab P., Xu G.-M., Wang B.-G. (2015). Rubrumazines A–C, indolediketopiperazines of the isoechinulin class from *Eurotium rubrum* MA-150, a fungus obtained from marine mangrove-derived rhizospheric soil. J. Nat. Prod..

[77] Guo W., Li D., Peng J., Zhu T., Gu Q., Li D. (2015). Penicitols A–C and penixanacid A from the mangrove-derived *Penicillium chrysogenum* HDN11-24. J. Nat. Prod..

[78] Fu P., MacMillan J.B. (2015). Thiasporines A–C, thiazine and thiazole derivatives from a marine-derived *Actinomycetospora chlora*. J. Nat. Prod..

[79] Schneider P., Misiek M., Hoffmeister D. (2008). In vivo and in vitro production options for fungal secondary metabolites. Mol. Pharm..

[80] Zhang H.W., Song Y.C., Tan R.X. (2006). Biology and chemistry of endophytes. Nat. Prod. Rep..

[81] Ludwig-Müller J. (2015). Plants and endophytes: Equal partners in secondary metabolite production?. Biotechnol. Lett..

[82] Katz L., Baltz R.H. (2016). Natural product discovery: Past, present, and future. J. Ind. Microbiol. Biotechnol..

[83] Tan R.X., Zou W.X. (2001). Endophytes: A rich source of functional metabolites. Nat. Prod. Rep..

[84] Deepika V.B., Murali T.S., Satyamoorthy K. (2016). Modulation of genetic clusters for synthesis of bioactive molecules in fungal endophytes: A review. Microbiol. Res..

[85] Daletos G., Ebrahim W., Ancheeva E., El-Neketi M., Lin WH., Proksch P., Grothaus P., Cragg G.M., Newman D.J. (2017). Microbial co-culture and OSMAC approach as strategies to induce cryptic fungal biogenetic gene clusters. Chemical Biology of Natural Products.

[86] Nützmann H.-W., Reyes-Dominguez Y., Scherlach K., Schroeckh V., Horn F., Gacek A., Schümann J., Hertweck C., Strauss J., Brakhage A.A. (2011). Bacteria-induced natural product formation in the fungus *Aspergillus nidulans* requires Saga/Ada-mediated histone acetylation. Proc. Natl. Acad. Sci. USA.

[87] Vasundhara M., Kumar A., Reddy M.S. (2016). Molecular approaches to screen bioactive compounds from endophytic fungi. Front. Microbiol..

[88] Kaul S., Sharma T., Dhar M.K. (2016). “Omics” tools for better understanding the plant–endophyte interactions. Front. Plant Sci..

[89] Ziemert N., Alanjary M., Weber T. (2016). The evolution of genome mining in microbes—A review. Nat. Prod. Rep..

[90] van der Lee T.A.J., Medema M.H. (2016). Computational strategies for genome-based natural product discovery and engineering in fungi. Fungal Genet. Biol..

[91] Pickens L.B., Tang Y., Chooi Y.-H. (2011). Metabolic engineering for the production of natural products. Annu. Rev. Chem. Biomol. Eng..

[92] Zhang M.M., Wang Y., Ang E.L., Zhao H. (2016). Engineering microbial hosts for production of bacterial natural products. Nat. Prod. Rep..

[93] Spencer A., Harrison S., Zonder J., Badros A., Laubach J., Bergin K., Khot A., Zimmerman T., Chauhan D., Levin N. (2018). A phase 1 clinical trial evaluating marizomib, pomalidomide and low-dose dexamethasone in relapsed and refractory multiple myeloma (NPI-0052-107): Final study results. Br. J. Haematol..

